# Adaptive Fuzzy Integral Sliding Mode Cooperative Control Based on Time-Delay Estimation for Free-Floating Close-Chain Manipulators

**DOI:** 10.3390/s24123718

**Published:** 2024-06-07

**Authors:** Zhongcan Li, Yufei Zhou, Mingchao Zhu, Qingwen Wu

**Affiliations:** 1 Changchun Institute of Optics, Fine Mechanics and Physics, Chinese Academy of Sciences, No. 3888, Dong Nanhu Road, Changchun 130033, China; lizhongcan20@mails.ucas.ac.cn (Z.L.); zhouyufei20@mails.ucas.ac.cn (Y.Z.); wuqw@ciomp.ac.cn (Q.W.); 2 University of Chinese Academy of Sciences, Beijing 100049, China

**Keywords:** time delay estimation, free-floating close-chain manipulators, adaptive fuzzy, integral sliding mode control, internal force

## Abstract

Space manipulators are expected to perform more challenging missions in on-orbit service (OOS) systems, but there are some unique characteristics that are not found on ground-based robots, such as dynamic coupling between space bases and manipulators, limited fuel supply, and working with unfixed bases. This paper focuses on trajectory-tracking control and internal force control for free-floating close-chain manipulators. First, the kinematics and dynamics of free-floating close-chain manipulators are given using the momentum conservation and spatial operator algebra (SOA) methodologies, respectively. Furthermore, an adaptive fuzzy integral sliding mode controller (AFISMC) based on time delay estimation (TDE) was designed for trajectory-tracking control, and a proportional-integral (PI) control strategy was adopted for internal force control. The global asymptotic stability of the proposed controller was proven by using the Lyapunov methodology. Three cases were conducted to verify the efficiency of the controller by using numerical simulations on two six-link manipulators with a free-floating base. The controller presents the desired tracking capability.

## 1. Introduction

In order to answer fundamental questions concerning human beings [1], such as “Is there life beyond earth?” and “What are the alternative sources of energy and materials in space?”, extremely large space structures, such as the James Webb Space Telescope [2] and space stations, need to be facilitated. With the limitations of space launch vehicles, on-orbit assembly technology can be used to assemble separate parts [3,4]. Roughly speaking, space missions are becoming increasingly complex, and space robots have significant advantages in tasks such as on-orbit assembly, repairs, and other high-risk service tasks [5]. Space robots are typically multi-rigid body systems connected by ideal joints. The current proposed concepts for space robots or manipulators include free-floating or flying space robot systems, macro-micro space robot systems, flexible structure-based space robot systems, and surface locomotive robot systems [6,7]. Due to fuel limitations, all reaction jets and wheels are turned off, leaving the space robots in a free-floating condition. However, the interaction force between the base and manipulators can disturb the attitude of the base, which, in turn, affects the position and posture of the end effectors. There are strong nonlinearities and dynamic coupling between the base and manipulators of a free-floating space robot system. Therefore, the mission planning and trajectory control of space robots is more complicated than fixed-base robots.

Compared with single-arm cases, the task capacity of dual-arm or multi-arm space robots in terms of flexibility and payload is significantly enhanced. However, free-floating space robotics systems are time-varying with strong nonlinearities and complex dynamics coupling between the manipulators and the base. Therefore, controller implementation for space robotics systems is more complicated than fix-base robotics systems or single-arm robotics systems. This complexity arises not only from the dynamic coupling between individual manipulator joints but also from between the manipulators themselves and between the manipulators and the free-floating base. Computed torque control (CTC) [8,9] is a common nonlinear control method that adopts nonlinear feedback to accurately offset the coupling terms and achieve decoupling. It then utilizes a proportional-derivative (PD) [10] controller to achieve trajectory tracking for a robot system. However, the control accuracy of this method heavily depends on the accuracy of the model. Additionally, obtaining accurate dynamics parameters of manipulators, such as the inertia matrix and Coriolis matrix, poses a significant challenge. The time delay estimation (TDE) technique provides another decoupling method that uses the acceleration and input torque of the previous moment to estimate the dynamic state of the current moment. Moreover, this method is a model-free decoupling method that is independent of the dynamics parameters of the manipulators’ system and has attracted strong interest from many researchers in the robot community [11,12,13,14,15]. first proposed an independent robot joint control method based on delay estimation in 1991 and verified the effectiveness of the algorithm based on the PUMA560 robot [16] presents a novel approach to improving trajectory tracking in autonomous underwater vehicles (AUVs) under the influence of disturbances. The approach combines an adaptive generalized super-twisting algorithm (AGSTA) controller with TDE. A novel robust control scheme was proposed in [17]. It combines a TDE with a backstepping control method and is designed to be independent of the robot’s dynamics model. Wang [18] utilized an incremental model predictive control (IMPC) scheme based on TDE for the constrained control of a manipulator. It must be noted, however, that the TDE error introduced by the delay estimation should be focused on to ensure the control accuracy of the robot system based on the discrete digital control system. Although the estimation error can be reduced by increasing the control frequency, the control frequency will be limited by the actual arithmetic power of the hardware. As is well known, sliding mode control (SMC) is insensitive to external disturbance, parameter perturbation, and model uncertainty, and it is widely used in nonlinear system control [19] proposed an adaptive variable structure controller (AVSC) that can outperform the smoothed quasi-continuous second-order SMC (SQC2S) in terms of the path tracking and set-point of dual-arm space robots, but the internal force control was not mentioned in this paper [20] proposed an adaptive neural network-based fast terminal sliding mode control scheme for the control of co-ordinated multiple mobile manipulators, the effectiveness of the controller was proved by simulation. Therefore, combining the SMC method with TDE has become the choice of many researchers [21,22,23,24]. That is, TDE is used to realize the decoupling of the robot system, and the sliding mode control is used to compensate for the various uncertainties, including the delay estimation error [21] adopted a terminal sliding mode control to compensate for the delay estimation errors [22] came up with an adaptive sliding mode control scheme by working with a pole-placement control method based on the TDE technique to achieve good tracking effects with low chattering for robot manipulators. Zhang [25] designed an adaptive robust controller based on the TDE technique and SMC. It was compared with a conventional CTC scheme and a CTC-based SMC scheme. In terms of control, when uncertainty and disturbances are imposed on the model, the adaptive robust controller’s performance based on the TDE technique and SMC can be maintained by reducing the time delay for space robots.

When multiple manipulators grasp a common target, closed kinematic chains are formed. The internal force will be induced between the target and the manipulators’ end effectors due to the kinematic constraints, i.e., pulling or pushing forces, on the target and the manipulators’ end effectors. For the flexible target body, the position of the center mass of the target will change, and the task will fail. The space robots and target may be damaged, or the manipulators will apply emergency braking for the rigid target when the internal force is out of control. Force-based control strategies are proposed for internal force control, and such schemes include impedance control [26] and proportional-integral (PI) control. Liu [27] adopted a PI controller to ensure that the internal force errors asymptotically converge to zero, and the stability of the PI controller was analyzed using the error dynamic equation.

In summary, the control of multi-arm close-chain manipulators is mainly divided into trajectory-tracking control and internal force control. Due to dynamic coupling, the control of free-floating close-chain manipulators is more complicated than single-arm space robots and fixed-base robots. Compared with the CTC control strategy, the SMC based on the TDE technique has better robustness and higher computational efficiency. The robustness of the SMC framework based on TDE is guaranteed using a larger tunable parameter. This tunable parameter is generally chosen as a sign function. The introduction of the sign function leads to discontinuities in the control system, which causes chattering effects in the control system. This chattering phenomenon is very serious for closed-chain multi-arm robot systems. As mentioned above, the kinematic constraints at the end of the manipulators generate internal forces. The sliding mode control-induced chattering effects of the system cause vibration at the end effector position, which destroys the kinematic constraints at the end of the manipulators and, thus, causes an abrupt change in the internal force. High-frequency bucket vibration can also cause fixture fatigue or failure of threaded fastener connections. Therefore, weakening the chattering effects of the sliding mode controller is meaningful and necessary for the motion control of multi-arm close-chain robots. Therefore, we propose a TDE-based adaptive fuzzy integral sliding mode control (AFISMC) in this paper to realize the dynamic decoupling and tracking control of free-floating close-chain manipulators.

The main contributions of this paper are listed as follows: (1) The SOA theory was used to model close-chain manipulators with free-floating bases. (2) The TDE method was used to realize system decoupling. In order to avoid the approaching stage of the classical sliding mode, the ISMC was proposed for the trajectory-tracking control of free-floating close-chain manipulators. (3) The adaptive fuzzy logical system was used to compensate for TDE error and alleviate the chattering effects caused by the ISMC, and the PI controller was used to accomplish internal force control.

The paper is organized as follows: The kinematics and dynamics of free-floating close-chain manipulators are introduced in Section 2. The decoupling controller design is investigated in Section 3. The main simulation results verify the efficiency of the controller in Section 4. Finally, conclusions are made in Section 5.

## 2. Math Modeling the Free-Floating Close-Chain Manipulators System

In order to save a ship’s fuel, the reaction control system of space robots will be turned off when docking in orbit to work. The space robots are in a free-floating condition. Therefore, the conservation of momentum is assumed, assuming no external forces and torques acting on the whole system. Before introducing the kinematic and dynamic formulations of free-floating closed-chain manipulators, the following basic assumptions are given:

1. The space robots and target are rigid bodies, and the robots’ joints are single-degree-of-freedom revolute joints;
2. The initial linear momentum and angular momentum of the free-floating space robot system are zero, and it is not affected by external forces and external moments;
3. There is no singular pose during manipulator motion, and the end effectors do not slide relative to the target.

### 2.1. Notation Definitions

**Figure 1:**
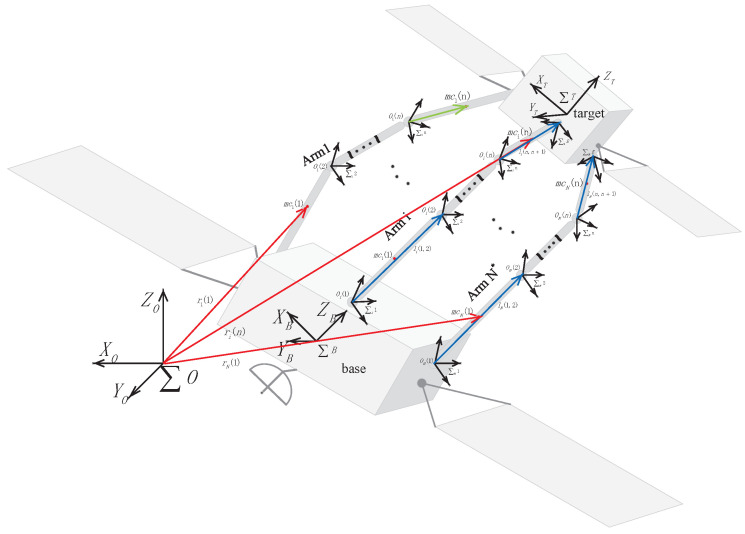
Structure diagram of free-floating close-chain manipulator system.

The free-floating close-chain manipulator system shown in Figure 1 consists of $N^{*}$ mechanical manipulators with *n* degrees of freedom, a base fixedly connected to the manipulators, and a target fixedly connected to the end effectors of the manipulators. The symbol of the space co-ordinate frame takes the form of that in [28]; in the absence of special instructions, all vectors and inertias are written independently of co-ordinates and are expressed in spatial frames. The links from the base to end effectors are numbered; the base is considered link 0, and the same rules apply to all the other manipulators. Subscript numbers are used to distinguish between different robotic manipulators. For instance, $m_{1}\left(2\right)$ represents the mass of link 2 on robot manipulator 1. It is assumed that all robot manipulators have the same number of links, and the spatial co-ordinate frame is attached to the axis of rotation of the links. The origin point of the spatial co-ordinate frame of link 2 on robot manipulator 1 is denoted by $O_{1}\left(2\right)$. Some important notations are superscript ‘*T*’ denotes the transpose of a matrix, and × denotes the cross-product of two vectors.




∑O

Inertial frame

∑B

Base frame

∑T

Target frame

$\sum_{i}K$

Spatial co-ordinate frame of link *k* on manipulator *i*

$\sum_{i}E$

End effector frame of link *k* on manipulator *i*$m_{t}$, $I_{t}$The mass and inertial tensor of the target$m_{i}\left(k\right)$, $I_{i}\left(k\right)$The mass and inertial tensor of link *k* on manipulator *i*

$O_{i}\left(k\right)$

The original point of the spatial co-ordinate frame of link k on manipulator *i*

$mc_{i}\left(k\right)$

The center of mass of link k on manipulator *i*

$p_{i}\left(k\right)$

The vector from $O_{i}\left(k\right)$ point to $mc_{i}\left(k\right)$

$r_{i}\left(k\right)$

The vector from the original point of the inertial frame point to the
center of mass of link *k* on manipulator *i* with respect to the inertial frame

$l_{i}\left(k,k+1\right)$

The spatial vector from $O_{i}\left(k\right)$ point to $O_{i}\left(k+1\right)$

$V_{i}\left(k\right)$

The spatial velocity of $O_{i}\left(k\right)$


$V_{i}\left(k\right)=\left[\begin{array}{c} v_{i}\left(k\right)\\ w_{i}\left(k\right) \end{array}\right]$



$\alpha_{i}\left(k\right)$

The spatial acceleration of $O_{i}\left(k\right)$


$\alpha_{i}\left(k\right)=\left[\begin{array}{c} {\dot{v}}_{i\left(k\right)}\\ {\dot{w}}_{i\left(k\right)} \end{array}\right]$



$f_{i}\left(k\right)$

The spatial force acting upon $O_{i}\left(k\right)$


$f_{i}\left(k\right)=\left[\begin{array}{c} F_{i}\left(k\right)\\ N_{i}\left(k\right) \end{array}\right]$



l×

The antisymmetric transformation of the vector, *l*


$l\times =\left[\begin{array}{ccc} 0 & -l\left(3\right) & l\left(2\right)\\ l\left(3\right) & 0 & -l\left(1\right)\\ -l\left(2\right) & l\left(1\right) & 0 \end{array}\right]$



$\phi_{i}\left(k+1,k\right)$

The linear operator that transforms the velocity from $O_{i}\left(k\right)$ to $O_{i}\left(k+1\right)$, Rk     k+1 denotes the rotation transformation matrix at $\sum_{i}K+1$ to $\sum_{i}K$. $0_{3}$ represents the zero matrix with a dimension of 3×3


ϕik+1,k=Rk     k+1−Rk     k+1lik,k+1×03Rk     k+1



$\psi_{i}\left(k\right)$

The linear operator that transforms the velocity from $O_{i}\left(k\right)$ to $O_{i}\left(k+1\right)$.
$E_{3}$ represents the unit matrix with a dimension of 3×3


$\psi_{i}\left(k\right)=\left[\begin{array}{cc} E_{3} & -p_{i}\left(k\right)\times \\ 0_{3} & E_{3} \end{array}\right]$



$a_{i}\left(k\right)$

The spatial bias acceleration of $O_{i}\left(k\right)$


$a_{i}\left(k\right)=\left[\begin{array}{c} w_{i}\left(k-1\right)\times \left(w_{i}\left(k-1\right)\times l_{i}\left(k-1,k\right)\right)\\ w_{i}\left(k-1\right)\times w_{i}\left(k\right) \end{array}\right]$



$b_{i}\left(k\right)$

The spatial bias force of $O_{i}\left(k\right)$


$b_{k}=\left[\begin{array}{c} m_{i}\left(k\right)w_{i}\left(k\right)\times \left(w_{i}\left(k\right)\times p_{i}\left(k\right)\right)\\ m_{i}\left(k\right)p_{i}\left(k\right)\times \left(w_{i}\left(k\right)\times \left(w_{i}\left(k\right)\times p_{i}\left(k\right)\right)\right)\\ +w_{i}\left(k\right)\times I_{i}\left(k\right)w_{i}\left(k\right) \end{array}\right]$



$H_{i}\left(k\right)$

The state vector of a joint for a rotational joint


$h_{i}\left(k\right)={\left[0,\;0,\;0,\;0,\;0,\;1\right]}^{T}$




### 2.2. Kinematics and Dynamics

The numerical simulation method can quickly and theoretically verify the feasibility of the control scheme and the reliability of the algorithm, greatly improving the design efficiency of the control scheme for the robot system. However, before conducting numerical simulation, it is necessary to first establish the mathematical model of the free-floating space robot system. In the following, the kinematics of a free-floating closed-chain manipulator are described using the algebraic theory of spatial operators using momentum conservation methods, and the generalized Jacobian matrix of the free-floating closed-chain manipulator system is computed. Immediately thereafter, the dynamics of the free-floating closed-chain manipulator system are described using spatial operator algebra using Newton-Euler methods.

**Figure 2:**
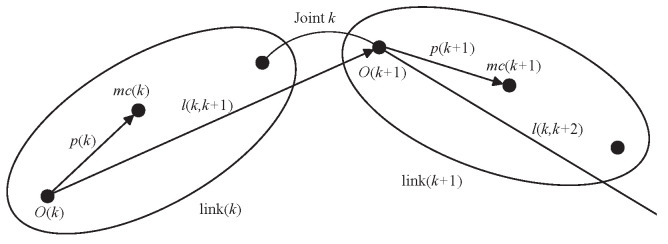
Motion relations of neighboring connecting links.

The SOA method involves defining the rigid body shift operator ϕ(k,k−1) to ensure that the velocity transfer equations, V(k)=ϕ(k,k−1)V(k−1), and for the transfer equations, $f\left(k\right)=\phi {\left(k,k-1\right)}^{T}f\left(k-1\right)$, of two neighboring rigid body links are satisfied. The relationship between two neighboring links is shown in Figure 2:

#### 2.2.1. Kinematics

The total linear momentum and angular momentum of the free-floating close-chain manipulator system can be expressed in the inertial frame:

(1a) $$\begin{array}{cc} P= & m\left(0\right)v^{c}\left(0\right)+\sum_{i=1}^{N^{*}}\sum_{k=1}^{n}m_{i}\left(k\right)v_{i}^{c}\left(k\right)+m_{t}v_{t}^{c} \end{array}$$

(1b) $$\begin{array}{cc} L= & I\left(0\right)w^{c}\left(0\right)+r\left(0\right)\times m\left(0\right)v^{c}\left(0\right)\\ \; & +\sum_{i=1}^{N^{*}}\sum_{k=1}^{n}\left(I_{i}\left(k\right)w_{i}^{c}\left(k\right)+r_{i}\left(k\right)\times m_{i}\left(k\right)v_{i}^{c}\left(k\right)\right)\\ \; & +I_{t}w_{t}^{c}+r_{t}\times m_{t}v_{t}^{c} \end{array}$$

where *P* and *L* are the linear momentum and angular momentum of the free-floating close-chain manipulator system, respectively, and $V\left(0\right)={\left[v^{T}\left(0\right),w^{T}\left(0\right)\right]}^{T}$ represents the velocity of the common base for all the manipulators. The superscript ‘*c*’ represents the center of mass. The velocity of the mass center can be obtained by multiplying a linear operator, ψ, such as $V^{c}\left(0\right)=\psi \left(0\right)V\left(0\right)$, and the velocity of other mass center points can be obtained by using a similar method. With the help of the SOA methodology, the velocity propagation of the common base to the end effectors may be written as

(2a) $$\begin{array}{cc} \; & V\left(0\right)=V_{1}\left(0\right)=\cdots =V_{N^{*}}\left(0\right) \end{array}$$

(2b) $$\begin{array}{cc} \; & V_{i}\left(k\right)=\phi_{i}\left(k,k-1\right)V_{i}\left(k-1\right)+h_{i}\left(k\right)\dot{\theta }\left(k\right)\;k=1\cdots n \end{array}$$

The velocity of end effectors:

(3) $$V_{i}\left(n+1\right)=\phi_{i}\left(n+1,n\right)V_{i}\left(n\right)$$

The velocity constraint between the target and end effectors may be written as

(4) $$J_{te}V_{t}=V\left(n+1\right)$$

where

(5a) $$\begin{array}{cc} \; & V_{t}={\left[\begin{array}{cc} v_{t}^{T} & w_{t}^{T} \end{array}\right]}^{T} \end{array}$$

(5b) $$\begin{array}{cc} \; & V\left(n+1\right)={\left[\begin{array}{ccc} V_{1}^{T}\left(n+1\right), & V_{2}^{T}\left(n+1\right),\cdots , & V_{N^{*}}^{T}\left(n+1\right) \end{array}\right]}^{T} \end{array}$$

(5c) $$\begin{array}{cc} \; & J_{te}={\left[\begin{array}{ccc} \phi_{1}^{-T}\left(t,n+1\right), & \phi_{2}^{-T}\left(t,n+1\right),\cdots , & \phi_{N^{*}}^{-T}\left(t,n+1\right) \end{array}\right]}^{T} \end{array}$$

where the subscript ‘*t*’ represents the target. $V_{t}$ represents the velocity of the target. $J_{te}$ is the Jacobian matrix between the target and end effectors.

Equations (2a), (2b), and (4) can be rewritten by using a Jacobian matrix as

(6) $$J_{te}V_{t}=J_{o}V\left(0\right)+J_{m}\dot{\theta }$$

where $J_{o}$ is the Jacobian matrix between the base and end effectors, and $J_{m}$ is the Jacobian matrix of the space manipulators. $\dot{\theta }={\left[\begin{array}{cccc} \dot{\theta_{1}^{T}}, & \dot{\theta_{2}^{T}}, & \cdots , & \dot{\theta_{N^{*}}^{T}} \end{array}\right]}^{T}$ represents the velocity vector of the joints. It can be seen from Equation (6) that the velocity of the target is related to the common base velocity and joint velocity of the space manipulators. Because of the dynamic coupling between the common base and space manipulators, the velocity of the target cannot be obtained directly via Equation (6). In order to overcome this problem, a dynamic equivalent manipulator has been proposed [29]. A generalized Jacobian matrix (GJM) was proposed by [30] to account for the dynamic interaction between the common base and space manipulators.

For free-floating close-chain manipulator system, which satisfies the law of conservation of momentum, according to assumption 2, the Equations (1a) and (1b) may be rewritten as

(7) $$\left[\begin{array}{c} P\\ L \end{array}\right]=H_{1}V\left(0\right)+H_{2}\dot{\theta }=0$$

By substituting Equation (7) into Equation (6), one can obtain

(8) $$J_{te}V_{t}=J_{g}\dot{\theta }$$

where the left of Equation (8) represents the velocity of the end effectors, $J_{g}=J_{m}-J_{o}H_{1}^{-1}H_{2}$ is the GJM. GJM directly relates the joints’ velocity to the end effectors’ velocity, and this brings convenience to mission trajectory planning [31], the controller design [32] of space robots, and learning impact dynamics when capturing a floating target in space [33]. The velocity of the base can be written as

(9) $$V\left(0\right)=J_{bm}\dot{\theta }$$

where $J_{bm}=-H_{1}^{-1}H_{2}$ is used to represent the velocity mapping of the space manipulators to the common base.

#### 2.2.2. Dynamics

Currently, the Newton-Euler, Lagrange, and Kane algorithms are mainly used for mechanical multibody dynamic modeling. These methods are not efficient enough; the numbers of the arithmetical operations of the Newton-Euler algorithm and Lagrange algorithm are $O\left(\beta^{3}\right)$, and the numbers of the arithmetical operations of the Kane algorithm are $O\left(\beta^{2}\right)$. When compared with the above three methods, the SOA algorithm using $O\left(\beta \right)$ is more suitable for a highly complex, free-floating close-chain manipulator system [34]. $\alpha \left(0\right)={\left[\begin{array}{cc} {\dot{v}}^{T}\left(0\right), & {\dot{w}}^{T}\left(0\right) \end{array}\right]}^{T}$ represents the acceleration of the common base for all the space manipulators. The acceleration propagation from the common base to the end effectors may be written by using SOA:

(10a) $$\begin{array}{cc} \; & \alpha \left(0\right)=\alpha_{1}\left(0\right)=\cdots =\alpha_{N}\left(0\right) \end{array}$$

(10b) $$\begin{array}{cc} \; & \alpha_{i}\left(k\right)=\phi_{i}\left(k,k-1\right)\alpha_{i}\left(k-1\right)+h_{i}\left(k\right){\ddot{\theta }}_{i}\left(k\right)+a_{i}\left(k\right)\;k=1\cdots n \end{array}$$

The acceleration of the end effectors:

(11) $$\alpha_{i}\left(n+1\right)=\phi_{i}\left(n+1,n\right)\alpha_{i}\left(n\right)+a_{i}\left(n+1\right)$$

With the help of a rigid body Newton-Euler Equation, the force propagation from the end effectors to the common base may be written by using SOA:

(12) $$\begin{array}{c} f_{i}\left(k\right)=\phi_{i}^{T}\left(k+1,k\right)f_{i}\left(k+1\right)+M_{i}\left(k\right)\alpha_{i}\left(k\right)+b_{i}\left(k\right)\;k=1\cdots n \end{array}$$

in Equation (12), the load assigned to the end effectors is expressed by $f_{i}\left(n+1\right)$. For rotational joints, the joint torques can be expressed as

(13) $$\tau_{i}\left(k\right)=h_{i}^{T}\left(k\right)f_{i}\left(k\right)$$

By combining Equations (10a)–(13), the dynamic formulation of a free-floating close-chain manipulator system can be expressed as follows:

(14) $$I\ddot{\theta }+C=\tau -J_{g}f\left(n+1\right)$$

where *I* is the generalized mass matrix of the space manipulators coupling with the common base. *C* is the matrix containing the centrifugal force and Coriolis force of the space manipulators and common base. τ denotes the torque vector of the space manipulators.

#### 2.2.3. Interaction with a Common Target

According to assumption 3, the closed kinematic chains are formed when the space manipulators hold a common target that is moving. The acceleration constraint equation between the end effectors and the target can be expressed as

(15) $$\dot{V}\left(n+1\right)={\dot{J}}_{g}\dot{\theta }+J_{g}\ddot{\theta }=J_{te}{\dot{V}}_{t}+a_{t}$$

where $a_{t}$ represents normal acceleration. The dynamic equation of the target can be written as

(16) $$M_{t}{\dot{V}}_{t}+b_{t}=J_{te}^{T}f\left(n+1\right)$$

#### 2.2.4. Internal Force Computation

Considering a free-floating close-chain manipulator system of $N^{*}$ manipulators rigidly grasping a common target, the total force imposed on the target can be decomposed into the motion, including the force and internal force. The total force on the target at the inertial frame is

(17) $$f_{t}=J_{te}^{T}f\left(n+1\right)=J_{te}^{T}\left(f_{E}+f_{I}\right)$$

where $f_{t}$ is the net force at the target’s center of mass, $f_{E}$ is the external force at the end effectors, and $f_{I}$ is the internal force at the end effectors.

In order to independently control the internal force and external force, $f\left(n+1\right)$ can be decomposed into $f_{E}$ and $f_{I}$. It is necessary to mention that an external force is mainly used to change the motion of the target. Internal force generates only tension or compression forces on the target. The physical meaning of internal force is that the sum of the virtual works done by the internal force $f_{I}$ for any and all virtual displacement of the object results in zero ($J_{te}^{T}f_{I}=0$) [35]. Furthermore, $f_{E}$ and $f_{I}$ can be written as

(18a) $$\begin{array}{cc} \; & f_{E}=J_{te}^{T+}J_{te}^{T}f\left(n+1\right) \end{array}$$

(18b) $$\begin{array}{cc} \; & f_{I}=\left(E_{6N^{*}}-J_{te}^{T+}J_{te}^{T}\right)f\left(n+1\right) \end{array}$$

where $J_{te}^{T+}$ is the inverse of $J_{te}^{T}$, $E_{6N^{*}}$ represents a unit matrix with a dimension of $6N^{*}$, $J_{te}^{T+}$ must take the following form in order for the moving component not to contribute to internal forces [36]:

(19a) $$\begin{array}{cc} \; & J_{te}^{T+}=\Xi J_{te}{\left(J_{te}^{T}\Xi J_{te}\right)}^{-1} \end{array}$$

(19b) $$\begin{array}{cc} \; & \Xi =\left[\begin{array}{ccc} \Theta & \cdots & 0_{6}\\ \vdots & \ddots & \vdots \\ 0_{6} & \cdots & \Theta \end{array}\right]\;with\;\Theta =\left[\begin{array}{cc} 0_{3} & E_{3}\\ E_{3} & 0_{3} \end{array}\right] \end{array}$$

#### 2.2.5. Summary

Considering joint friction torque and a model with uncertainties, and when combining Equations (13)–(15), the dynamic formulation of a free-floating close-chain manipulator system can be rewritten as follows:

(20) $$H\ddot{\theta }+N=\tau -J_{g}^{T}f_{I}-\tau_{f}-\tau_{d}$$

where

(21a) $$\begin{array}{cc} \; & H=I+J_{g}^{T}J_{te}^{T+}M_{t}J_{te}^{+}J_{g} \end{array}$$

(21b) $$\begin{array}{cc} \; & N=C+J_{g}^{T}J_{te}^{T+}\left(M_{t}J_{te}^{+}{\dot{J}}_{g}\dot{\theta }-M_{t}J_{te}^{+}a_{t}+b_{t}\right) \end{array}$$

$\tau_{f}$ is the frictional torque, and $\tau_{d}$ is the error torque caused by a non-nominal model. The internal force computation can be viewed in Equations (17)–(19b). In the next section, a robust tracking control method for free-floating closed-chain manipulators based on TDE decoupling is proposed based on the above mathematical model.

## 3. Controller Design

The control tasks of a free-floating close-chain manipulator system are divided into two parts: (1) trajectory tracking and (2) internal force control. In this section, the TDE-based ISMC controller is designed for free-floating close-chain manipulator trajectory-tracking control, and the classical PI controller is used to control the internal force between the manipulators.

Equation (20) can be divided into a trajectory control part and an internal force control part, as in [37]

(22a) $$\begin{array}{cc} \; & H\ddot{\theta }+N=\tau_{p}-\tau_{f}-\tau_{d} \end{array}$$

(22b) $$\begin{array}{cc} \; & 0=\tau_{I}-J_{g}^{T}f_{I} \end{array}$$

where $\tau_{p}+\tau_{I}=\tau$. $\tau_{p}$ is the trajectory control torque, and $\tau_{I}$ is the internal force control torque.

### 3.1. TDE Method

By introducing a positive definite diagonal matrix, $\bar{H}$, another expression of Equation (22a) can be rewritten as follows:

(23) $$\bar{H}\ddot{\theta }+\bar{N}=\tau_{p}$$

which includes all uncertainties and nonlinear terms:

(24) $$\bar{N}=\left(H-\bar{H}\right)\ddot{\theta }+N+J_{g}^{T}f_{E}+\tau_{f}+\tau_{d}$$

In order to estimate $\bar{N}$, TDE was used so that, for a given time, *t*, the nonlinear terms ${\bar{N}}_{\left(t\right)}$ at time *t* can be estimated by using $\hat{N}$ with a sufficiently small sampling period, δ, where the following holds: $\lim_{\delta \to 0}{\bar{N}}_{\left(t-\delta \right)}={\bar{N}}_{\left(t\right)}$. Let $\hat{N}$ indicate the estimate of $\bar{N}$; then, by using $\hat{N}={\hat{N}}_{\left(t-\delta \right)}$ from Equation (23), we can obtain

(25) $$\hat{N}=\tau_{p(t-\delta )}-\bar{H}{\ddot{\theta }}_{\left(t-\delta \right)}$$

Let $\Delta =\bar{N}-\hat{N}$ denote the TDE error.

**Lemma 1.**  *The TDE error *Δ* is bounded [26] if the positive definite diagonal matrix $\bar{H}$ is selected to satisfy the following condition:*

(26) $$∥E_{6N}-H^{-1}\bar{H}∥\leq 1$$

Equation (22a) can be modified by using the TDE methodology as follows:
  (27a) $$\begin{array}{cc} \; & \bar{H}\ddot{\theta }+\hat{N}+\Delta =\tau_{p} \end{array}$$
  (27b) $$\begin{array}{cc} \; & \hat{N}\approx \bar{N} \end{array}$$

### 3.2. AFISMC

TDE is an effective method for realizing dynamically decoupled control for free-floating close-chain manipulators. However, the control accuracy of the controller will be reduced due to the introduction of the delay estimation error. Sliding mode control is robust to factors such as external perturbations to the control system, parameter perturbation, and model uncertainty. In order to compensate for the TDE error caused by TDE and improve the robustness of the controller simultaneously, the ISMC was used. Compared with the classical sliding mode surface, the integral sliding mode omits the reaching phase, and it can enter the sliding mode directly by selecting the appropriate initial values.

First, the integral sliding mode surface can be designed as follows:

(28a) $$\begin{array}{cc} \; & s=s_{o}+z \end{array}$$

(28b) $$\begin{array}{cc} \; & s_{o}=\dot{e}+\Lambda e \end{array}$$

(28c) $$\begin{array}{cc} \; & \dot{z}=-\Lambda \dot{e}+K_{1}\dot{e}+K_{2}e\;\mathrm{with}\;z_{\left(0\right)}=-\Lambda e_{\left(0\right)}-{\dot{e}}_{\left(0\right)} \end{array}$$

where $s_{o}$ is the classic sliding surface, *z* is the integral term, and $z_{\left(0\right)}$ is initial value of *z*. $e=\theta_{d}-\theta$ denotes the joint position error vector, and $\dot{e}={\dot{\theta }}_{d}-\dot{\theta }$ denotes the joint velocity error vector. $\Lambda ,\;K_{1},\;K_{2}$ are the gain matrices of the corresponding signal. After the integration of Equation (28c), the integral sliding mode surface can be obtained:

(29) $$s=\dot{e}+K_{1}e+K_{2}\int_{0}^{t}ed\xi -K_{1}e_{\left(0\right)}-{\dot{e}}_{\left(0\right)}$$

**Figure 3:**
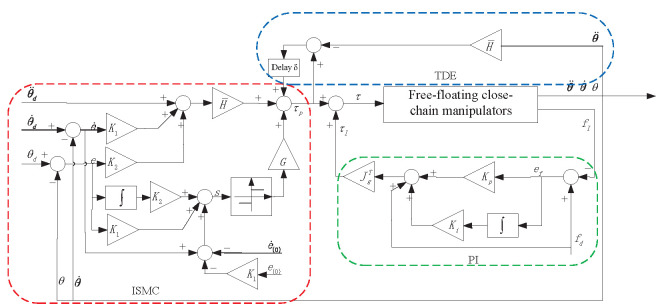
TDE-based ISMC with a sign function controlling strategy.

The assumption is that we can always find a positive definite matrix that satisfies Lemma 1. In that case, the TDE-based control law for the ISMC with a symbol switching function can be written as follows:

(30) $$\tau_{p}=\bar{H}\left({\ddot{\theta }}_{d}+K_{1}\dot{e}+K_{2}e\right)+\hat{N}+Gsgn\left(s\right)$$

where ${\ddot{\theta }}_{d}$ denotes the ideal joint acceleration of the manipulators, $G=daig\left[G_{1},G_{2},\cdots ,G_{N^{*}}\right]$ is diagonal positive definite switching gain matrix to compensate for the error caused by a non-nominal model and joint friction. Assuming all the unknown terms are bounded, we can always find a positive definite diagonal matrix, *G*, that satisfies $G_{i}>\Delta_{i}$, and sgn(·) is a sign function. The TDE-based ISMC with the sign function controlling strategy is illustrated in Figure 3.

The stability of this control law can be proved by selecting the following Lyapunov function:

(31) $$V=\frac{1}{2}s^{T}s$$

by differentiating Equation (31) with respect to time and substituting Equation (30) into it:

(32) $$\begin{array}{cc} \dot{V} & =s^{T}\dot{s}\\ \; & =s^{T}\left({\ddot{\theta }}_{d}-\ddot{\theta }+K_{1}\dot{e}+K_{2}e\right)\\ \; & =s^{T}\left({\ddot{\theta }}_{d}-{\bar{H}}^{-1}\left(\tau_{p}-\hat{N}-\Delta \right)+K_{1}\dot{e}+K_{2}e\right)\\ \; & =s^{T}\left({\ddot{\theta }}_{d}-{\bar{H}}^{-1}\left(\bar{H}\left({\ddot{\theta }}_{d}+K_{1}\dot{e}+K_{2}e\right)+Gsgn\left(s\right)-\Delta \right)\right.\\ \; & \;\;\left.+K_{1}\dot{e}+K_{2}e\right)\\ \; & =s^{T}{\bar{H}}^{-1}\left(\Delta -Gsgn(s)\right)\leq 0 \end{array}$$

thus, stability is proven.

The characteristics of the SMC depend only on the designed hyperplane and are not related to uncertainties; therefore, sliding mode control is highly robust. However, introducing the switching function leads to discontinuity in the control system, resulting in strong chattering effects. The chattering of the sliding surfaces causes the end effector’s trajectory to oscillate, and it induces chattering effects in the internal force. The chattering effect of the internal force leads to periodic contact stress and fatigue damage. In order to mitigate the chattering effect, several effective methods have been proposed, such as the boundary layer method [26], high-order SMC [38,39], the fuzzy method [40,41], and adaptively optimized parameters [42].

**Lemma 2**  ([43])**.** *Given any real continuous function*,* $f\left(s\right)$*,* defined on a bounded compact region*,* $\Omega_{s}$*,* and for any positive constant*,* ς*,* there always exists a fuzzy logic system*,* $\hat{f}\left(s|r\right)$*,* to satisfy.*

(33) $$\exists \varsigma >0\;\underset{s\in \Omega_{s}}{\sup}\left|f\left(s\right)-\hat{f}\left(s|r\right)\right|\leq \varsigma$$

According to Lemma 1, the delay estimation error is bounded when $\bar{H}$ satisfies Equation (26). Lemma 2 shows that the output, $\hat{f}\left(s|r\right)$, of a fuzzy logic system can come close to any continuous bounded function. Therefore, a fuzzy logic system can not only weaken the chattering effect of the sliding mode controller but also compensate for the delay estimation error, and in order to improve the adaptive ability of the system at the same time, three steps are needed to construct the fuzzy logic system based on the adaptive rate (suppose the free-floating close-chain manipulator system consists of two manipulators with n degrees of freedom):

Step 1

Define $m_{i}$ fuzzy sets $\pi_{i}^{l_{i}}\left(l_{i}=1,2,\cdots ,m_{i}\right)$ for variable $s_{i}\left(i=1,2,\cdots ,2n\right)$;

Step 2

The following $\Gamma (\Gamma =\prod_{i=1}^{2n}m_{i})$ fuzzy rules are used to construct the fuzzy logical system:

Rule(γ) : IF $s_{1}$ is $\pi_{1}^{\gamma_{1}}$ and $s_{2}$ is $\pi_{2}^{\gamma_{2}}$ and ⋯ and $s_{2n}$ is $\pi_{2n}^{\gamma_{2n}}$, THEN $\hat{f}\left(s|r\right)$ is $E^{\gamma }$.

$s_{i}$ denotes the inputs of the fuzzy logical system, and the output, $\hat{f}\left(s|r\right)$, of the fuzzy logical system denotes the approximation of an unknown continuous function, f(s). By using the singleton fuzzifier, product inference, and the center of the average defuzzifier, the output of the fuzzy logical system can be expressed:

(34) $$\hat{f}\left(s|r\right)=\frac{\sum_{\gamma =1}^{\Gamma }r_{\gamma }\prod_{i=1}^{2n}\mu_{\pi_{i}^{\gamma_{i}}}\left(s_{i}\right)}{\sum_{\gamma =1}^{\Gamma }\prod_{i=1}^{2n}\mu_{\pi_{i}^{\gamma_{i}}}\left(s_{i}\right)}$$

in Equation (34), $\mu_{\pi_{i}^{\gamma_{i}}}\left(s_{i}\right)$ represents the membership function of variable $s_{i}$. The membership function is one of the key points of a fuzzy logic system. In the process of constructing a fuzzy logic system, the choice of the membership function relies entirely on expert experience. However, for nonlinear systems with multiple degrees of freedom, Gaussian and bell-shaped membership functions are more useful and robust than triangular and trapezoidal membership functions. In this paper, the membership function is designed as a Gaussian function:

(35) $$\mu_{\pi }\left(s_{i}\right)=\exp\left[\frac{-{\left(s_{i}-\mu_{0}\right)}^{2}}{\sigma^{2}}\right]$$

where $r_{\gamma }$ is a free parameter. By defining the fuzzy basic function as $p\left(s_{i}\right)=\prod_{i=1}^{2n}\mu_{\pi_{i}^{\gamma_{i}}}\left(s_{i}\right)/\sum_{\gamma =1}^{\Gamma }\prod_{i=1}^{2n}\mu_{\pi_{i}^{\gamma_{i}}}\left(s_{i}\right)$, Equation (34) can be rewritten as

(36) $$\hat{f}\left(s|r\right)=r^{T}P\left(s\right)$$

where $r^{T}=\left[r_{1},r_{2},\cdots ,r_{\Gamma }\right]$ represents the vector of the free parameters, and $P\left(s\right)={\left[p_{1}\left(s\right),p_{2}\left(s\right),\cdots ,p_{\Gamma }\left(s\right)\right]}^{T}$ denotes the vector of fuzzy basic functions.

Step 3

In general, the vector of a free parameter can be determined by expert experience, and it does not change during the whole control process. Therefore, it can change according to the adaptive rate to improve the adaptive ability of a fuzzy logic system. The adaptive rate is designed as

(37) $${\dot{r}}_{i}=s_{i}P_{i}\left(s_{i}\right)$$

By combining Equations (30), (34) and (35), the control law for the AFISMC with TDE can be written as follows:

(38a) $$\begin{array}{cc} \; & \tau_{p}=\bar{H}\left({\ddot{\theta }}_{d}+K_{1}\dot{e}+K_{2}e\right)+\hat{N}+\Lambda_{1}\hat{f}\left(s|r\right)+\Lambda_{2}s \end{array}$$

(38b) $$\begin{array}{cc} \; & {\dot{r}}_{i}=s_{i}P_{i}\left(s_{i}\right) \end{array}$$

where $\Lambda_{1}$ and $\Lambda_{2}$ are diagonal positive definite matrices. By combining this with Equation (22b) and the PI control strategy, the internal force control rate can be expressed as

(39) $$\tau_{I}=J_{g}\left(f_{Id}+K_{p}e_{f}+K_{i}\int_{0}^{t}e_{f}d\xi \right)$$

where $e_{f}$ denotes the error of the internal force. $K_{p}$ and $K_{i}$ are the gain matrices of the corresponding signal, which are diagonally positive definite. Therefore, by combining the trajectory control law and internal force control law, the control input of the free-floating close-chain manipulator system can be expressed as

(40) $$\tau =\tau_{p}+\tau_{I}$$

**Figure 4:**
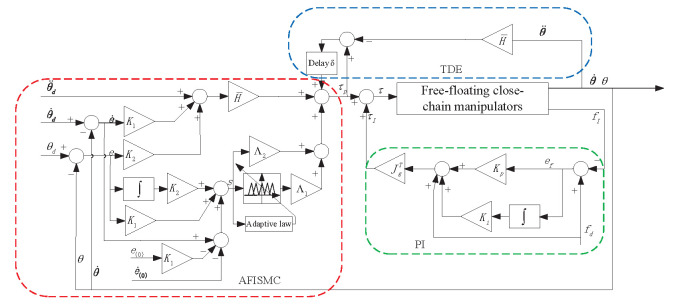
TDE-based AFISMC controlling strategy.

The TDE-based AFISMC with a PI internal force controller is illustrated in Figure 4.

**Proof.**  The Lyapunov function is chosen as

(41) $$V=V_{1}+V_{2}+V_{3}$$

where $V_{1}=1/2e_{f}^{T}e_{f}$, $V_{2}=1/2s^{T}s$, $V_{3}=1/2\left(\delta r^{T}\Lambda_{1}{\bar{H}}^{-1}\delta r\right)$, $\delta r=r_{d}-r$ denotes the difference between the ideal parameters and actual parameters. Differentiating $V_{1}$ and ${\dot{V}}_{1}=e_{f}^{T}{\dot{e}}_{f}$ by substituting Equation (39) into the Equation (22b), yields

(42) $$K_{p}e_{f}+K_{i}\int_{0}^{t}e_{f}d\xi =0$$

When differentiating Equation (42), the ${\dot{e}}_{f}$ can be written as ${\dot{e}}_{f}=-K_{p}^{-1}K_{i}e_{f}$; $K_{p}$ and $K_{i}$ are diagonally positive definite; therefore, ${\dot{V}}_{1}=-e_{f}^{T}K_{p}^{-1}K_{i}e_{f}\leq 0$. Differentiating $V_{2}$ and $V_{3}$ yields

(43) $$\begin{array}{cc} {\dot{V}}_{2}+{\dot{V}}_{3} & =s^{T}\dot{s}+\sum_{i=1}^{2n}\frac{\Lambda_{1i}}{{\bar{H}}_{i}}\delta r_{i}\delta {\dot{r}}_{i}\\ \; & =s^{T}\left(\ddot{e}+K_{1}\dot{e}+K_{2}e\right)+\sum_{i=1}^{2n}\frac{\Lambda_{1i}}{{\bar{H}}_{i}}\delta r_{i}\delta {\dot{r}}_{i}\\ \; & =s^{T}\left(\ddot{\theta_{d}}-\ddot{\theta }+K_{1}\dot{e}+K_{2}e\right)+\sum_{i=1}^{2n}\frac{\Lambda_{1i}}{{\bar{H}}_{i}}\delta r_{i}\delta {\dot{r}}_{i} \end{array}$$

${\bar{H}}_{i}$ is the i−th element on the diagonal of $\bar{H}$, and $\Lambda_{1i}$ is the ith element in the diagonal of $\Lambda_{1}$. By substituting Equation (38a) into Equation (27a), we obtain

(44) $$\ddot{\theta }={\ddot{\theta }}_{d}+K_{1}\dot{e}+K_{2}e+{\bar{H}}^{-1}\left(\Lambda_{1}\hat{f}\left(s|r\right)+\Lambda_{2}s-\Delta \right)$$

By replacing Equation (44) into the Equation (43), we obtain

(45) $$\begin{array}{cc} {\dot{V}}_{2}+{\dot{V}}_{3} & =s^{T}{\bar{H}}^{-1}\left(\Delta -\Lambda_{1}\hat{f}\left(s|r\right)+\Lambda_{2}s\right)+\sum_{i=1}^{2n}\frac{\Lambda_{1i}}{{\bar{H}}_{i}}\delta r_{i}\delta {\dot{r}}_{i}\\ \; & =\sum_{i=1}^{2n}\frac{\Lambda_{1i}s_{i}}{{\bar{H}}_{i}}\left(\Delta_{i}-{\hat{f}}_{i}\left(s_{i}|r_{i}\right)\right)+\sum_{i=1}^{2n}\frac{\Lambda_{1i}}{{\bar{H}}_{i}}\delta r_{i}\delta {\dot{r}}_{i}-s^{T}{\bar{H}}^{-1}\Lambda_{2}s\\ \; & =\sum_{i=1}^{2n}\frac{\Lambda_{1i}s_{i}}{{\bar{H}}_{i}}\left(\Delta_{i}-r_{i}P_{i}\left(s_{i}\right)\right)+\sum_{i=1}^{2n}\frac{\Lambda_{1i}}{{\bar{H}}_{i}}\delta r_{i}\delta {\dot{r}}_{i}-s^{T}{\bar{H}}^{-1}\Lambda_{2}s \end{array}$$

By replacing $\delta r_{i}=r_{di}-r_{i}$ and $\delta {\dot{r}}_{i}=-\dot{r_{i}}$ into Equation (45), and then combining this with Equation (38b), Equation (45) can be rewritten as

(46) $$\begin{array}{cc} {\dot{V}}_{2}+{\dot{V}}_{3} & =\sum_{i=1}^{2n}\frac{\Lambda_{1i}s_{i}}{{\bar{H}}_{i}}\left(\Delta_{i}-r_{i}P_{i}\left(s_{i}\right)\right)\\ \; & -\sum_{i=1}^{2n}\frac{\Lambda_{1i}s_{i}}{{\bar{H}}_{i}}\left(r_{di}-r_{i}\right)P_{i}\left(s_{i}\right)-s^{T}{\bar{H}}^{-1}\Lambda_{2}s\\ \; & =\sum_{i=1}^{2n}\frac{\Lambda_{1i}s_{i}}{{\bar{H}}_{i}}\left(\Delta_{i}-r_{di}P_{i}\left(s_{i}\right)\right)-s^{T}{\bar{H}}^{-1}\Lambda_{2}s \end{array}$$

According to Lemma 2,

(47) $$\exists \varsigma_{i}\geq 0,\;\left|\Delta_{i}-r_{di}P_{i}\left(s_{i}\right)\right|\leq \varsigma_{i}=\zeta_{i}\left|s_{i}\right|$$

Therefore, Equation (46) can be expressed as follows:

(48) $$\begin{array}{cc} {\dot{V}}_{2}+{\dot{V}}_{3} & =\sum_{i=1}^{2n}\frac{\Lambda_{1i}s_{i}}{{\bar{H}}_{i}}\left(\Delta_{i}-r_{di}P_{i}\left(s_{i}\right)\right)-s^{T}{\bar{H}}^{-1}\Lambda_{2}s\\ \; & \leq \sum_{i=1}^{2n}\frac{\Lambda_{1i}s_{i}}{{\bar{H}}_{i}}\zeta_{i}\left|s_{i}\right|-\sum_{i=1}^{2n}\frac{\Lambda_{2i}}{{\bar{H}}_{i}}s_{i}^{2}\\ \; & \leq \sum_{i=1}^{2n}\frac{\Lambda_{1i}\zeta_{i}-\Lambda_{2i}}{{\bar{H}}_{i}}s_{i}^{2}\leq 0 \end{array}$$

where $\Lambda_{2i}$ is the ith element on the diagonal of $\Lambda_{2}$ and $\Lambda_{2i}\geq \Lambda_{1i}\zeta_{i}$; above all,

(49) $$\begin{array}{cc} \dot{V} & ={\dot{V}}_{1}+{\dot{V}}_{2}+{\dot{V}}_{3}\\ \; & \leq -e_{f}^{T}K_{p}^{-1}K_{i}e_{f}+\sum_{i=1}^{2n}\frac{\Lambda_{1i}\zeta_{i}-\Lambda_{2i}}{{\bar{H}}_{i}}s_{i}^{2}\leq 0 \end{array}$$

Thus, stability is proven. □

## 4. Simulation Experiments

**Figure 5:**
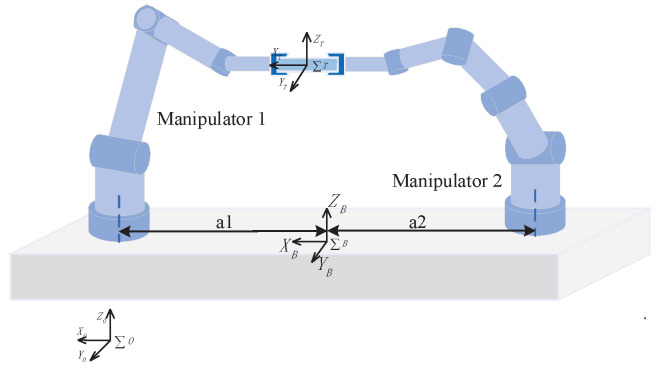
A co-operative close-chain manipulator system schematic.

In order to demonstrate the effectiveness of the proposed control scheme (Figure 4), numerical simulations were carried out on two of the same six-link manipulators co-operatively carrying a common target, as shown in Figure 5, through three cases: (1) the nominal model, (2) the model with uncertainties, frictions, and disturbances, (3) the model with different delay time length.Additionally, to verify the compensation capability of the adaptive fuzzy logic system, following the above two case studies, a study on different delay time lengths was also conducted. At the same time, the ability to weaken the chattering was verified by comparing the control method proposed in this paper with the integral sliding mode method combined with the sign function and saturation function.

**Figure 6:**
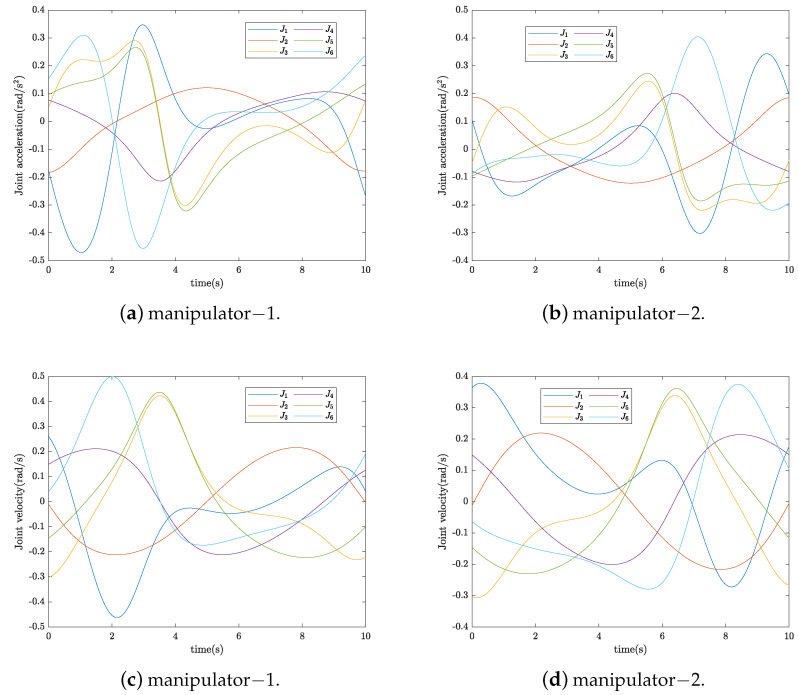

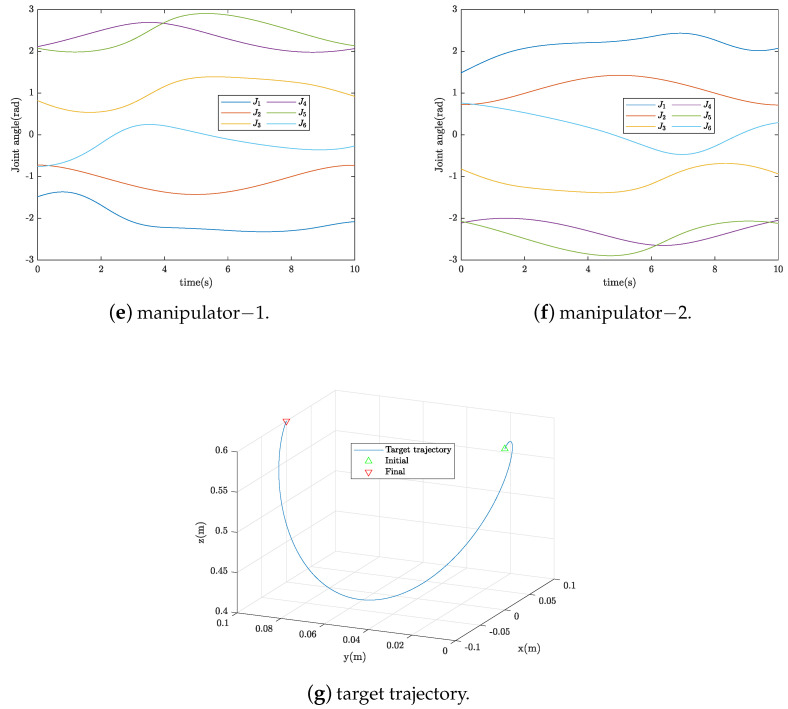
Planned joint trajectories and target trajectory.

As shown in Figure 5, two six-degrees-of-freedom manipulators were attached to a free-floating base, and both manipulators co-operate to carry a common target. The base frame is located at the center of mass of the base and between the two manipulators with an equal distance of a1=a2=0.5 m from each of them. The mass of the base m(0)=500 (kg), and the inertia matrix $I\left(0\right)=diag\left[200,200,200\right]$ (kg · m^2^). Both manipulators need to co-operate to hold a target, the mass of which $m_{t}=3$ (kg) and the inertia matrix of which $I_{t}=diag\left[0.1,0.1,0.1\right]$ (kg · m^2^). The initial position and attitude of the base and target are the same as the inertial frame. The D-H parameters of the manipulators are given in Table 1, and the dynamic parameters are given in Table 2. Where $\left({\left(\right)}_{k}^{i}\right)$ denotes the parameter that is defined in link k frame on manipulator i. The last column in Table 1 represents the initial joints’ angles for the manipulators. It is assumed that the ideal trajectory of the target is a spiral line, defined as follows:

(50) $$x\left(t\right)=\left[\begin{array}{c} 0.1sin(2t)\\ 0.01t\\ 0.1cos(2t)+0.5 \end{array}\right]$$

in which $x{\left(t\right)}^{T}\in R^{3}$ denotes the reference trajectory vector of the target. The reference contact internal force was chosen as $f_{1I}={\left[-20,0,0,0,0,0\right]}^{T}\left(N\right)$ and $f_{2I}={\left[20,0,0,0,0,0\right]}^{T}\left(N\right)$ for manipulator-1 and manipulator-2, respectively. According to the kinematics formulation, the joint trajectory can be calculated by using the inverse kinematics method and using Equations (8) and (9), as shown in Figure 6. The sampling time was set at 1 ms, and the terminal time was set as t=10 s.

### 4.1. Case A: The Nominal Model

**Figure 7:**
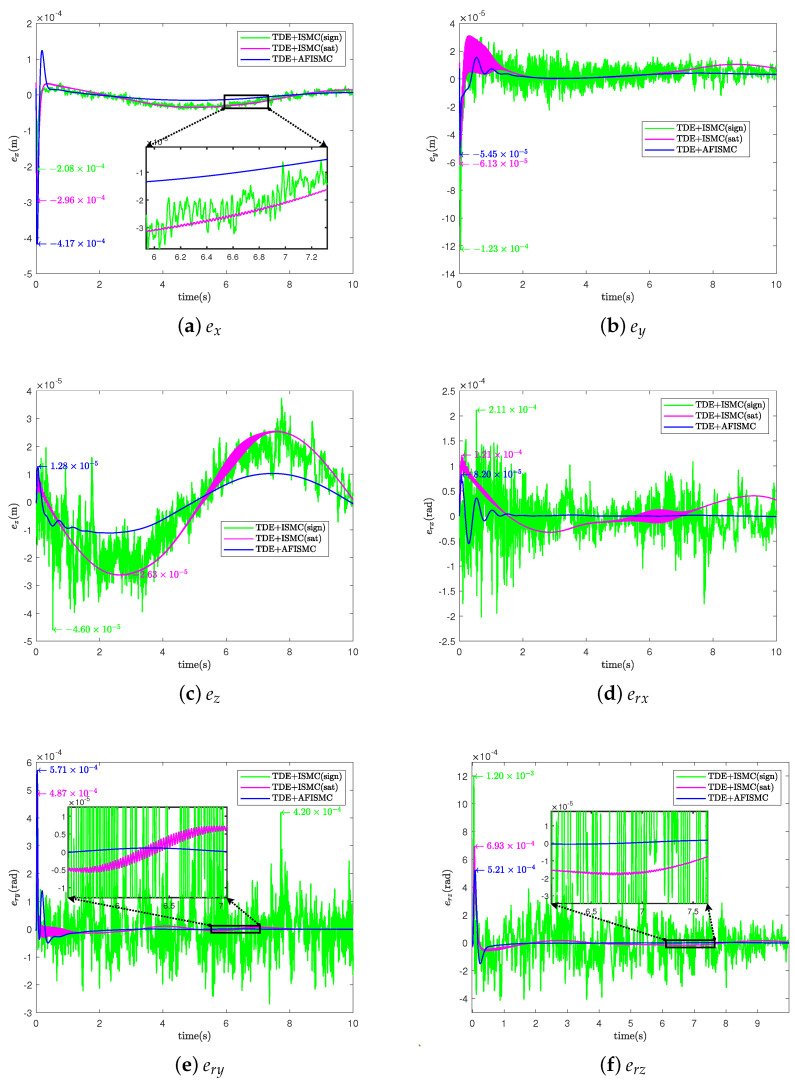
Object’s trajectory -tracking errors for case A.

**Figure 8:**
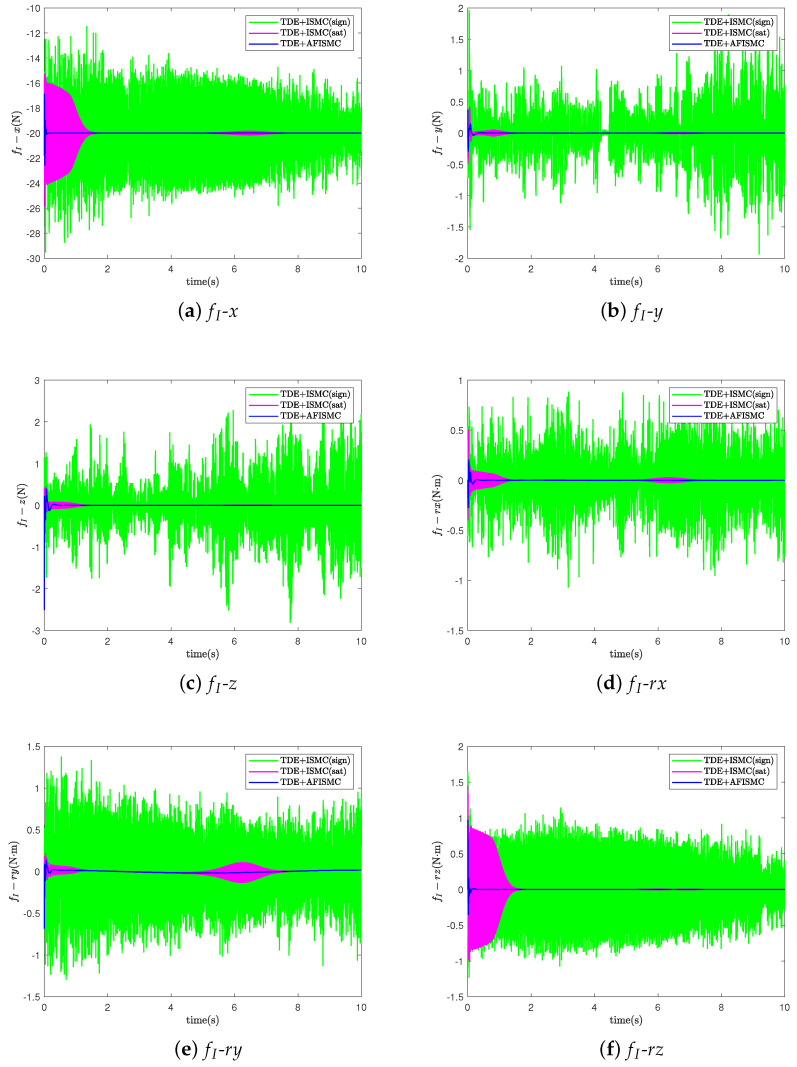
The internal force for case A.

**Figure 9:**
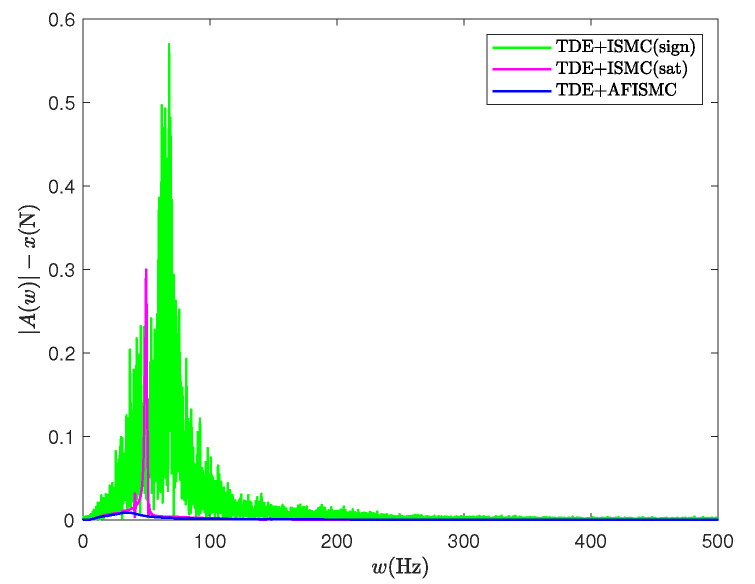
The internal force in the x-direction with FFT for case A.

In this case, the nominal model is used to evaluate the performance of the controller that we propose (Figure 4). For the TD-based AFISMC controller, the constant diagonal matrix, $\bar{H}$, the delay time length, δ, the expected joint velocity feedback gain matrix, $K_{1}$, the expected joint position feedback gain matrix, $K_{2}$, and the adaptive fuzzy logical system matrices $\Lambda_{1}$ and $\Lambda_{2}$ needed to be determined. After many setups, the parameter matrices can be viewed in Table 3. Figure 7 shows the object’s trajectory-tracking errors, and Figure 8 depicts the control performance of the internal force. r(·) indicates the direction of rotation around the (·)-axis. In Figure 7, the numeric text indicates the maximum overshoot, and the numeric color indicates the control method (consistent with the legend). Observing Figure 7 reveals that the maximum overshoot of AFISMC is smaller than the sign function versus the saturation function in most directions, and the steady state error is smaller. In addition, during the error convergence process, the tracking error obtained using the AFISMC method has less chattering effect and smaller vibration amplitude. In this paper, the mean and root mean square of the trajectory-tracking error were chosen to evaluate the tracking accuracy of the control algorithm, and the mean and root mean square of the error can be defined as

(51a) $$\begin{array}{cc} \; & e_{mean}=\frac{1}{N_{s}}\sum_{i=1}^{N_{s}}\left(e(i)\right) \end{array}$$

(51b) $$\begin{array}{cc} \; & {∥e∥}_{rms}=\sqrt{\frac{1}{N_{s}}\sum_{i=1}^{N_{s}}{∥e(i)∥}^{2}} \end{array}$$

where $e_{mean}$ is the mean of the control accuracy error. ${∥e∥}_{rms}$ is the root mean square of the control accuracy error. e(i) is the trajectory-tracking error at the *i*th sampling, and $N_{s}$ is the whole number of the sampling times. Table 4 and Table 5 show the mean and root mean square (RMS) values of the trajectory-tracking errors obtained via the different control methods, respectively. By looking at the computational results in Table 4 and Table 5, it can be seen that the tracking error of the AFISMC-based controller is smaller than the remaining two methods in almost all directions. The internal force is shown in Figure 8. Although all methods ensure internal force stabilization, it is evident—even through the time-domain signals—that the AFISMC method controls a smoother internal force. In order to more easily analyze the chattering effect of the internal force, we performed a fast Fourier transform (FFT) of the internal force error signal (in the x-direction, for example), and the result is depicted in Figure 9. When combined with the results of the FFT of the internal force error, the adaptive fuzzy method can suppress the chattering effect more effectively, especially in the high-frequency part.

### 4.2. Case B: The Model with Uncertainties, Frictions, and Disturbances

In order to approximate the actual physical model more closely, some uncertainties, such as joint friction and inertial parameter measurement errors, are taken into account on the basis of the nominal model. Joint friction moments were modeled using a linear friction model and a nonlinear friction model, respectively, so that the joint friction moment could be expressed by the following equation:

(52a) $$\begin{array}{cc} \; & \tau_{f1}=0.1\dot{\theta } \end{array}$$

(52b) $$\begin{array}{cc} \; & \tau_{f2}=f_{v}\dot{\theta }+\left[f_{c}+f_{cs}sech\left(\beta \dot{\theta }\right)tanh\left(\alpha \dot{\theta }\right)\right] \end{array}$$

where $\tau_{f}$ denotes the friction torques. Equation (52a) describes a linear friction model. Equation (52b) describes a nonlinear friction model. In Equation (52b), $f_{v}=0.00986$ indicates the coefficient of viscous friction, $f_{c}=0.743$ is the Coulomb friction factor, $f_{cs}=3.99$ and α=3.24 are used to construct the Stribeck friction action, and β=0.799 ensures that the equation is continuous when the relative sliding velocity is zero [44]. The exact inertial parameters of the robotic arm are difficult to obtain accurately, and even if they are obtained through software calculations, they can be biased during the assembly process. Therefore, the mass of the robotic arm linkage was adjusted to $m_{1}=4.5$ (kg), $m_{2}=5.5$ (kg), $m_{3}=4.5$ (kg), $m_{4}=4$ (kg), $m_{5}=3$ (kg), and $m_{6}=3$ (kg). The center of mass of the linkage of the robotic arm was adjusted to be $p_{1,2}\left(1\right)=\left[0\;m;0\;m;-0.15\;m\right]$, $p_{1,2}\left(3\right)=\left[0\;m;0\;m;-0.18\;m\right]$, $p_{1,2}\left(5\right)=\left[0\;m;0\;m;-0.15\;m\right]$. If the robotic arms are handling a flexible object or a liquid container, then the center of mass of the target object will also change. This causes the end loads to transform. With the help of the GJM, load perturbation variation can be translated into the robot joint space and considered as joint disturbance torques. The disturbance torques are assumed as follows:

(53) $$\begin{array}{cc} \; & \tau_{d1}^{1}=\tau_{d2}^{1}=\tau_{d3}^{1}=0.03\left(sin\left(2\pi t\right)+sin\left(0.5\pi t\right)\right)\\ \; & \tau_{d4}^{1}=\tau_{d5}^{1}=\tau_{d6}^{1}=0.02\left(sin\left(2\pi t\right)+sin\left(0.5\pi t\right)\right)\\ \; & \tau_{d1}^{2}=\tau_{d2}^{2}=\tau_{d3}^{2}=0.03\left(sin\left(2\pi t\right)+sin\left(0.5\pi t\right)\right)\\ \; & \tau_{d4}^{2}=\tau_{d5}^{2}=\tau_{d6}^{2}=0.02\left(sin\left(2\pi t\right)+sin\left(0.5\pi t\right)\right) \end{array}$$

**Figure 10:**
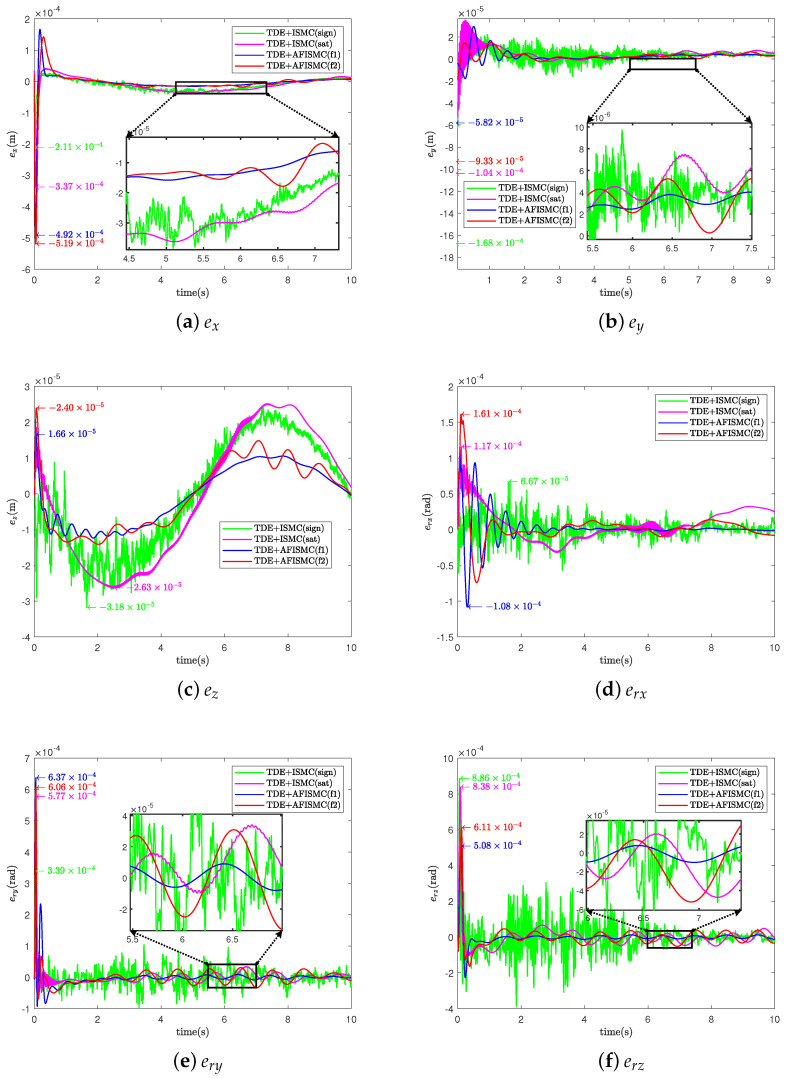
Object’s trajectory−tracking errors for case B.

**Figure 11:**
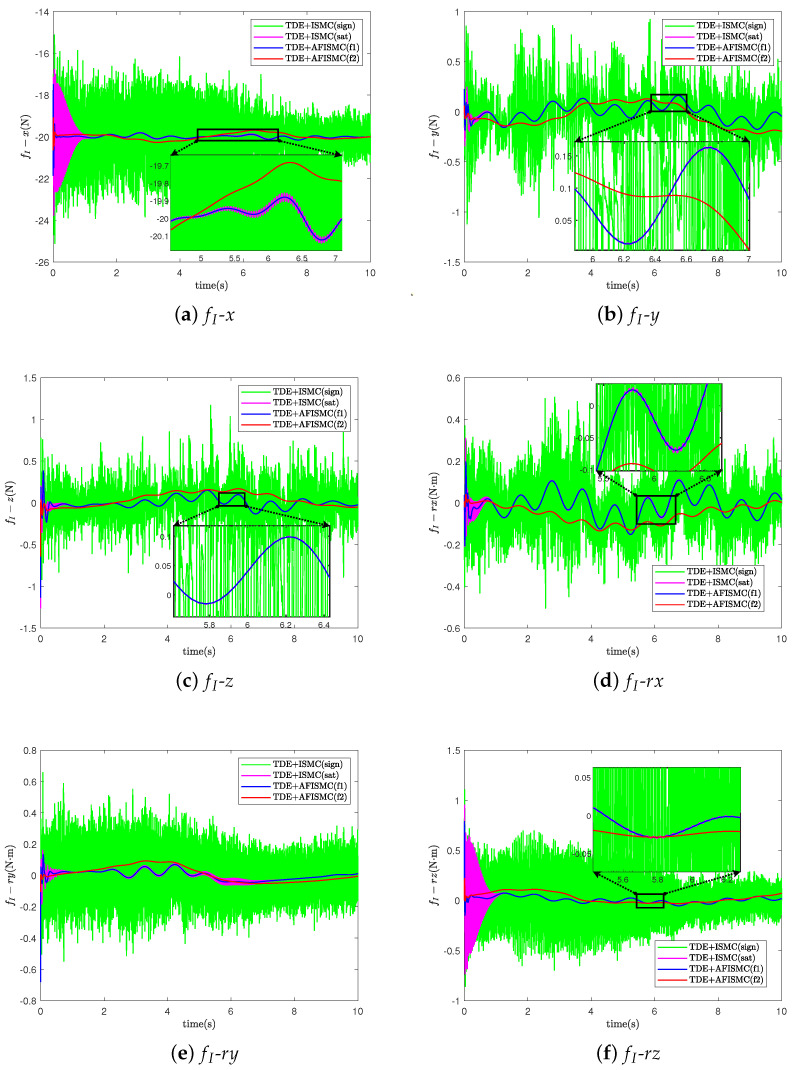
The internal force for case B.

**Figure 12:**
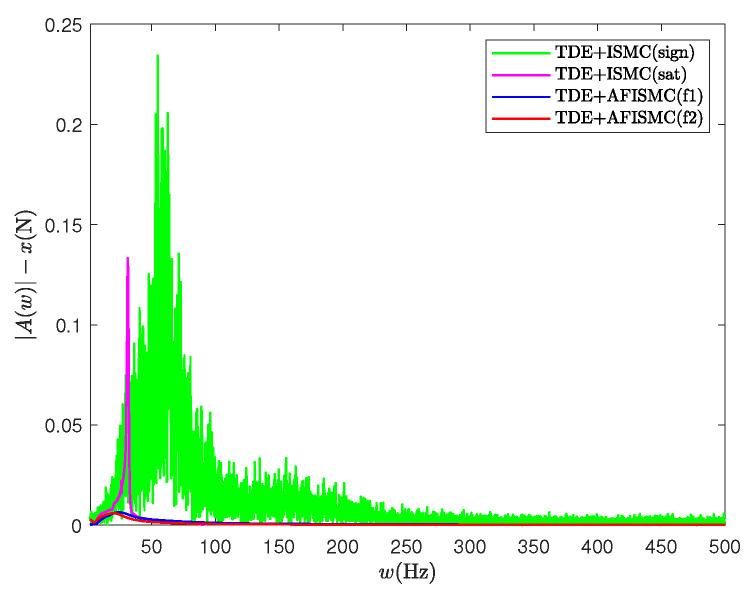
The internal force in the x-direction with FFT for case B.

For the TDE-based AFISMC controller, the signals gain matrix of $K_{1}$, $K_{2}$, $\Lambda_{2}$, and $K_{p}$, $K_{i}$ are the same as in case A. $\Lambda_{1}$ was set as $\Lambda_{1}=diag\left[400,400,\cdots ,400\right]$. The performance of trajectory-tracking control is shown in Figure 10, and the internal force control performance is depicted in Figure 11. TDE + IMSC (sign) and TDE + IMSC (sat) experiments were undertaken only using the linear friction models, i.e., Equation (52a). The mean and root mean square of the trajectory-tracking errors are also calculated in Table 6 and Table 7. According to Figure 12, the AFISMC based on TDE also still has a good ability to weaken the internal chattering phenomenon under the disturbance of uncertainties and perturbations.

### 4.3. Case C: The TDE-Based AFISMC with Different Delay Time Length

**Figure 13:**
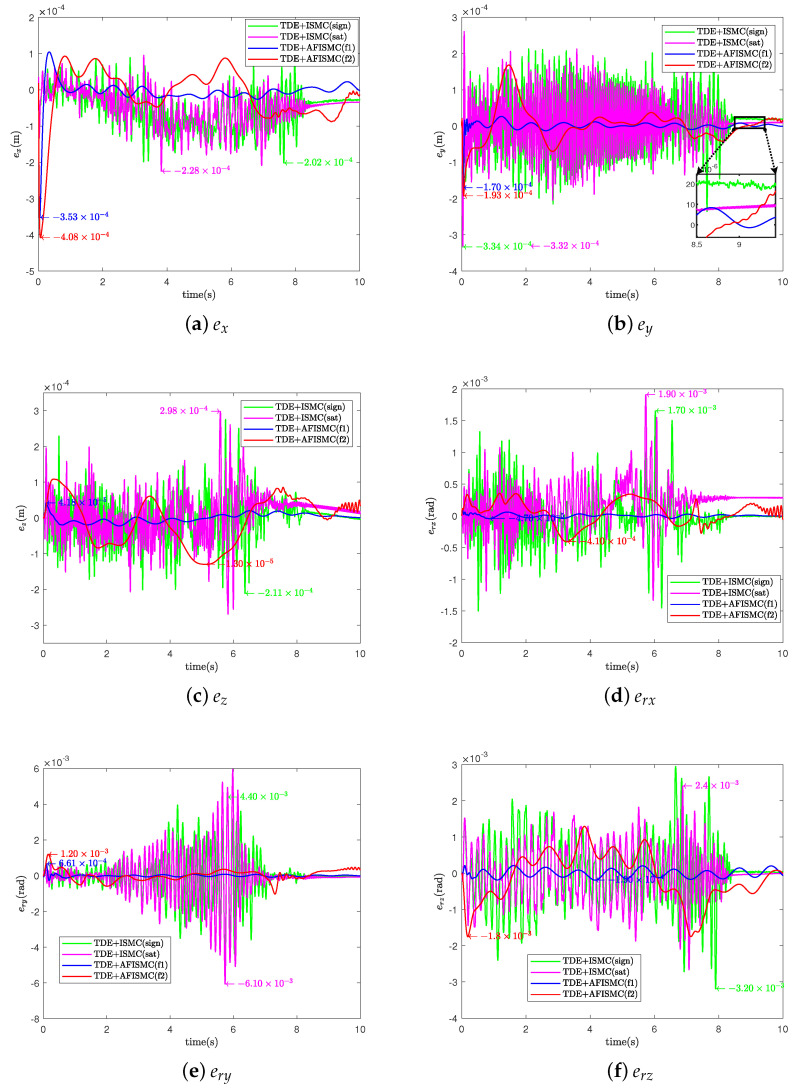
Object’s trajectory−tracking errors for case C (δ=3 ms).

**Figure 14:**
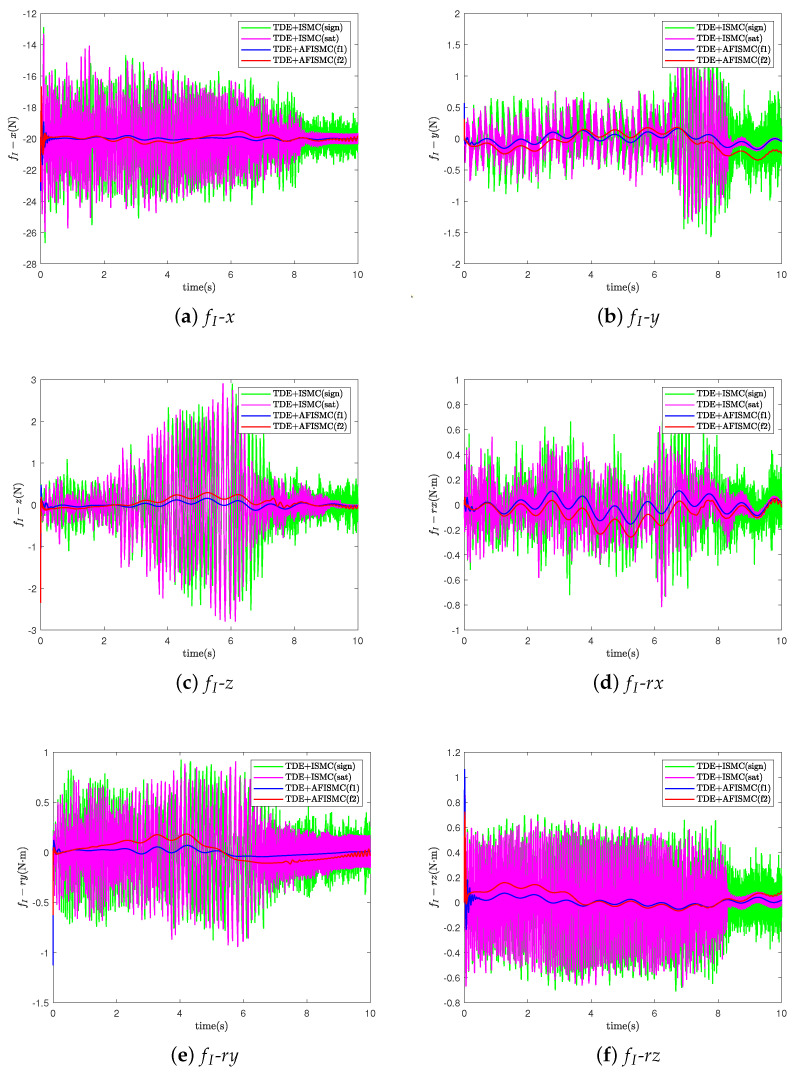
The internal force for case C (δ=3 ms).

In the above two cases, the control performance with a sampling period of δ=1 ms was tested. The length of the time delay has a great influence on the control accuracy; the smaller the delay time, the smaller the delay estimation error, and the fuzzy logic system more easily compensates for the delay error; therefore, the control accuracy is higher. In this case, the different delay time lengths, δ, where δ=3 ms and δ=5 ms, were used to verify the compensation capability of the adaptive fuzzy logic system, where the uncertainties, frictions, and disturbances are the same as in case B. The parameter $\Lambda_{1}$ was adjusted to $\Lambda_{1}=\left[60,60,\cdots ,60\right]$, $\Lambda_{2}$ was adjusted to $\Lambda_{2}=\left[0.5,0.5,\cdots ,0.5\right]$, $K_{i}$ was adjusted to $K_{i}=\left[200,200,\cdots ,200\right]$, and $K_{p}$ was adjusted to $K_{p}=\left[5,5,\cdots ,5\right]$. $K_{1}$ and $K_{2}$ are three times larger than those in case B. The performance of trajectory-tracking control is shown in Figure 13, and the internal force control performance is depicted in Figure 14.

**Figure 15:**
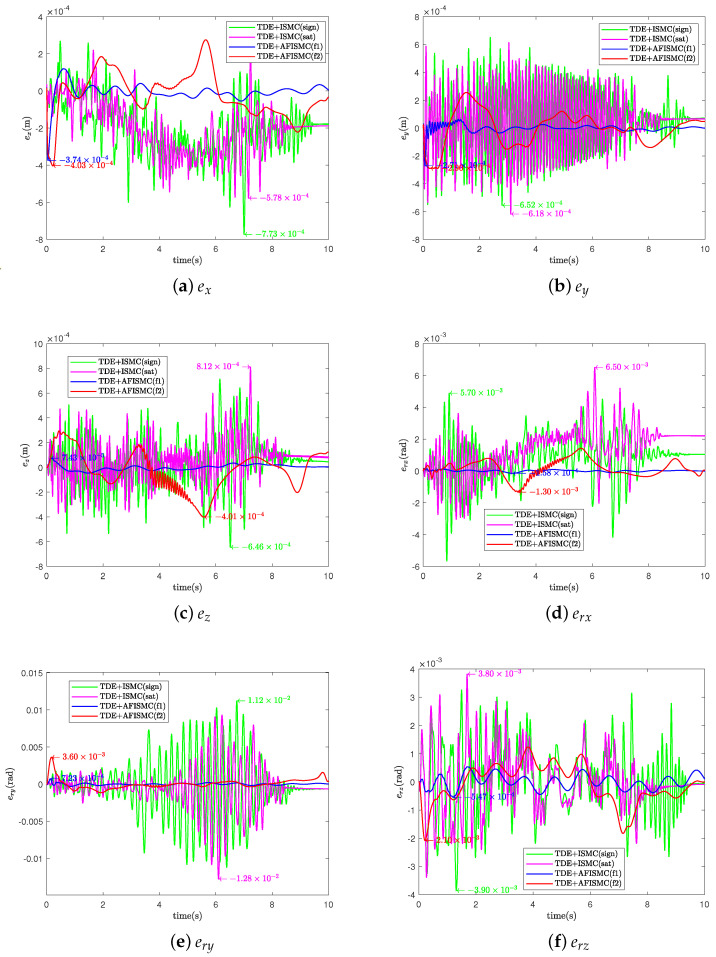
Object’s trajectory−tracking errors for case C (δ=5 ms).

**Figure 16:**
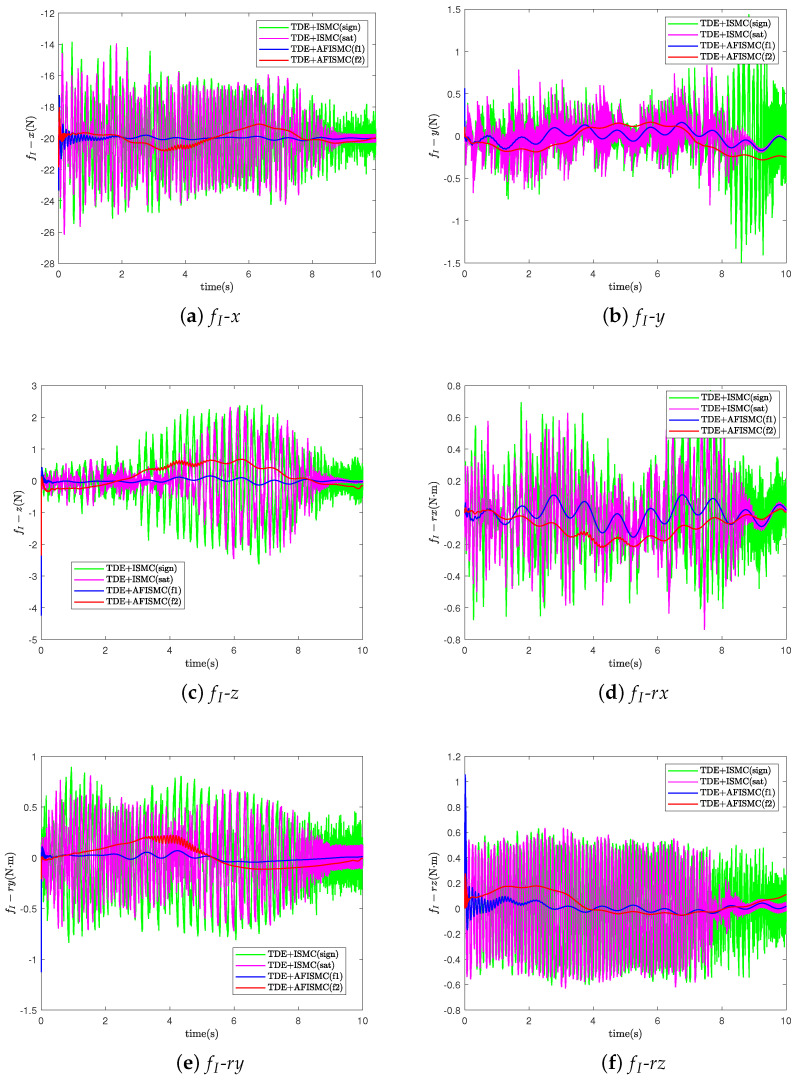
The internal force for case C (δ=5 ms).

Figure 15 and Figure 16 show the simulation results of the trajectory-tracking error and internal force control at a delay time of δ=5 ms, respectively.

The mean and root mean square of the trajectory-tracking errors for different delay times were simultaneously calculated and are shown in Table 8, Table 9, Table 10 and Table 11.

### 4.4. Discussion

The robustness and compensation ability of the AFISMC controller were respectively verified through simulation experiments in different cases. By calculating the mean and root mean square of the errors, it can be observed that the control accuracy and disturbance-resistance ability of the AFISMC controller are higher than those of the other two methods, effectively mitigating the chattering effect caused by the integral sliding mode control. In Case 3, the delay time δ was set to three times and five times that of the simulation-solving step. However, upon observing the data in Table 8, Table 9, Table 10 and Table 11, it can be seen that the control accuracy was not significantly reduced and is much higher than that of the other two methods. This demonstrates the effective compensation ability of the adaptive fuzzy logic system. If the nonlinear joint friction model is used, the control method proposed in this paper is less effective compared to the linear model. However, when compared to the performance of the other two methods for the linear model, the method proposed in this paper still demonstrates high control accuracy and effectively reduces the system chattering effect. By observing the time-domain curves of the internal forces, it is evident that replacing the sign function and saturation function with the adaptive fuzzy logic system effectively reduces the high-frequency chattering and oscillation amplitude of the internal forces. Although the saturated function also weakens the unsteady shaking to some extent, a closer examination of the local zoomed-in graphs in the internal force control results reveals that the use of the saturated function also introduces a small, high-frequency oscillation to the internal force.

## 5. Conclusions

In this paper, the adaptive fuzzy internal sliding model based on a TDE state feedback control strategy is proposed for trajectory-tracking control, and a proportional-integral control strategy was adopted for the internal force control of free-floating close-chain manipulators with model uncertainties and frictions. In the proposed control strategy, TDE is adopted for dynamic decoupling between the free-floating base and manipulators. Moreover, an integral sliding mode control strategy combined with TDE is used to improve the robustness of the system. The adaptive fuzzy logical system is adopted instead of a symbol-switching function to degrade the chattering effect of the trajectory and internal force. In order to track the desired internal forces that are in the null space of the Jacobian matrix between the end effectors and a common target, a proportional integral is used. Finally, three cases were studied regarding the two six-link manipulators co-operatively carrying a common target with a free-floating base by using numerical simulation. The simulation results indicate that both the tracking errors of trajectory and the internal force can converge to small values by setting proper gain parameter matrices. Our future work will be aimed at applying the controller to a microgravity simulation system to show its effectiveness and practicability.

## Figures and Tables

**Table 1:** D-H parameters of the space manipulators.

Manipulator-i	Link No.	$\alpha_{k-1}^{i}$ (deg)	$a_{k-1}^{i}$ (m)	$d_{k-1}^{i}$ (m)	$\theta_{k-1}^{i}$ (deg)
1/2	1	0/0	0.5/−0.5	0.34/0.34	−85.07/85.07
	2	90/90	0/0	0/0	−41.45/41.45
	3	−90/−90	0/0	0.35/0.35	46.90/−46.90
	4	90/90	0/0	0/0	121.10/−121.10
	5	−90/−90	0/0	0.32/0.32	118.90/−118.90
	6	90/90	0/0	0/0	−43.63/43.63
	End-effectors	–/–	0/0	–/–	–/–

**Table 2:** Dynamic parameters of the space manipulators represented in the link frame.

Parameter	Manipulator-1/2					
	Link 1	Link 2	Link 3	Link 4	Link 5	Link 6
m(kg)	4.86	5.81	4.60	4.48	2.88	3.21
$I_{xx}\left(\mathrm{kg}\cdot {\mathrm{mm}}^{2}\right)$	32,252.69	28,425.08	30,469.76	18,758.07	13,962.24	11,564.95
$I_{yy}\left(\mathrm{kg}\cdot {\mathrm{mm}}^{2}\right)$	37,556.12	67,502.04	34,675.90	45,962.17	16,999.11	27,325.70
$I_{zz}\left(\mathrm{kg}\cdot {\mathrm{mm}}^{2}\right)$	14,566.83	50,269.06	12,841.42	33,596.43	6975.97	19,550.87
$I_{xy}\left(\mathrm{kg}\cdot {\mathrm{mm}}^{2}\right)$	60.07	35.11	54.8034	107.97	−47.80	74.16
$I_{xz}\left(\mathrm{kg}\cdot {\mathrm{mm}}^{2}\right)$	−1157.77	−7310.82	−1068.84	−5987.53	−713.73	−3458.24
$I_{yz}\left(\mathrm{kg}\cdot {\mathrm{mm}}^{2}\right)$	22.80	−21.77	27.89	−4.26	−22.00	−17.96
$p_{x}\left(m\right)$	0	0	0	0	0	0
$p_{y}\left(m\right)$	0	0	0	0	0	0
$p_{z}\left(m\right)$	−0.183	0	−0.178	0	−0.160	0

**Table 3:** Controller parameters.

Parameters	Values
$\bar{H}$	$\bar{H}=diag\left[\bar{h},\bar{h}\right],\;\mathrm{where}\;\bar{h}=diag\left[0.26,0.57,0.15,0.25,0.02,0.02\right]$
$K_{1}$	$K_{1}=diag\left[20,30,20,20,20,20,20,30,20,20,20,20\right]$
$K_{2}$	$K_{2}=diag\left[25,35,25,25,25,25,25,35,25,25,25,25\right]$
$\Lambda_{1}$	$\Lambda_{1}=diag\left[360,400,360,360,360,360,360,400,360,360,360,360\right]$
$\Lambda_{2}$	$\Lambda_{2}=diag\left[3,3,3,3,3,3,3,3,3,3,3,3\right]$
$K_{p}$	$K_{p}=diag\left[10,10,10,10,10,10,10,10,10,10,10,10\right]$
$K_{i}$	$K_{i}=diag\left[100,100,100,100,100,100,100,100,100,100,100,100\right]$
*G*	$G=diag\left[3,3,3,3,3,3,3,3,3,3,3,3\right]$

**Table 4:** The mean of the control accuracy errors of the target trajectory for case A.

Method	$e_{mean}-x$	$e_{mean}-y$	$e_{mean}-z$	$e_{mean}-rx$	$e_{mean}-ry$	$e_{mean}-rz$
TDE + ISMC (sign)	$1.08\times 10^{-5}$	$3.37\times 10^{-6}$	$-4.63\times 10^{-7}$	$-3.68\times 10^{-6}$	$-1.46\times 10^{-6}$	$-1.43\times 10^{-6}$
TDE + ISMC (sat)	$1.08\times 10^{-5}$	$5.26\times 10^{-6}$	$-5.40\times 10^{-7}$	$-8.46\times 10^{-6}$	$-3.59\times 10^{-7}$	$-9.24\times 10^{-7}$
TDE + AFISMC	$-4.79\times 10^{-6}$	$2.50\times 10^{-6}$	$-4.38\times 10^{-7}$	$1.23\times 10^{-6}$	$-4.51\times 10^{-7}$	$-4.46\times 10^{-7}$

**Table 5:** The RMS of the control accuracy errors of the target trajectory for case A.

Method	$e_{rms}-x$	$e_{rms}-y$	$e_{rms}-z$	$e_{rms}-rx$	$e_{rms}-ry$	$e_{rms}-rz$
TDE + ISMC (sign)	$2.27\times 10^{-5}$	$9.97\times 10^{-6}$	$1.62\times 10^{-5}$	$3.93\times 10^{-5}$	$8.44\times 10^{-5}$	$1.42\times 10^{-4}$
TDE + ISMC (sat)	$3.00\times 10^{-5}$	$8.31\times 10^{-6}$	$1.82\times 10^{-5}$	$3.11\times 10^{-5}$	$2.11\times 10^{-5}$	$5.06\times 10^{-5}$
TDE + AFISMC	$3.36\times 10^{-5}$	$4.70\times 10^{-6}$	$7.97\times 10^{-6}$	$1.05\times 10^{-5}$	$3.15\times 10^{-5}$	$4.57\times 10^{-5}$

**Table 6:** The mean of the control accuracy errors of the target trajectory for case B.

Method	$e_{mean}-x$	$e_{mean}-y$	$e_{mean}-z$	$e_{mean}-rx$	$e_{mean}-ry$	$e_{mean}-rz$
TDE + ISMC (sign)	$1.06\times 10^{-5}$	$3.02\times 10^{-6}$	$-6.55\times 10^{-7}$	$-1.40\times 10^{-6}$	$-1.67\times 10^{-6}$	$-9.17\times 10^{-7}$
TDE + ISMC (sat)	$-1.10\times 10^{-5}$	$5.28\times 10^{-6}$	$-5.90\times 10^{-7}$	$-8.37\times 10^{-6}$	$-4.33\times 10^{-7}$	$-2.06\times 10^{-6}$
TDE + AFISMC (f1)	$-4.87\times 10^{-6}$	$2.43\times 10^{-6}$	$-5.15\times 10^{-7}$	$1.34\times 10^{-6}$	$-4.02\times 10^{-7}$	$-5.28\times 10^{-7}$
TDE + AFISMC (f2)	$-4.93\times 10^{-6}$	$2.49\times 10^{-6}$	$-4.59\times 10^{-7}$	$3.33\times 10^{-6}$	$-1.08\times 10^{-6}$	$-1.12\times 10^{-6}$

**Table 7:** The RMS of the control accuracy errors of the target trajectory for case B.

Method	$e_{rms}-x$	$e_{rms}-y$	$e_{rms}-z$	$e_{rms}-rx$	$e_{rms}-ry$	$e_{rms}-rz$
TDE + ISMC (sign)	$2.21\times 10^{-5}$	$1.18\times 10^{-5}$	$1.52\times 10^{-5}$	$3.27\times 10^{-5}$	$3.05\times 10^{-5}$	$9.44\times 10^{-5}$
TDE + ISMC (sat)	$3.30\times 10^{-5}$	$8.62\times 10^{-6}$	$1.80\times 10^{-5}$	$2.44\times 10^{-5}$	$2.98\times 10^{-5}$	$7.16\times 10^{-5}$
TDE + AFISMC (f1)	$4.07\times 10^{-5}$	$6.01\times 10^{-6}$	$8.22\times 10^{-6}$	$2.08\times 10^{-5}$	$4.09\times 10^{-5}$	$4.96\times 10^{-5}$
TDE + AFISMC (f2)	$4.71\times 10^{-5}$	$6.81\times 10^{-6}$	$9.55\times 10^{-6}$	$2.59\times 10^{-5}$	$6.71\times 10^{-5}$	$6.05\times 10^{-5}$

**Table 8:** The mean of the control accuracy errors of the target trajectory for case C (δ=3 ms).

Method	$e_{mean}-x$	$e_{mean}-y$	$e_{mean}-z$	$e_{mean}-rx$	$e_{mean}-ry$	$e_{mean}-rz$
TDE + ISMC (sign)	$-5.08\times 10^{-5}$	$1.24\times 10^{-5}$	$-3.14\times 10^{-5}$	$-3.16\times 10^{-5}$	$-2.18\times 10^{-5}$	$1.09\times 10^{-5}$
TDE + ISMC (sat)	$-4.83\times 10^{-5}$	$7.23\times 10^{-6}$	$1.09\times 10^{-5}$	$1.94\times 10^{-4}$	$-6.45\times 10^{-5}$	$7.61\times 10^{-5}$
TDE + AFISMC (f1)	$-5.01\times 10^{-6}$	$1.00\times 10^{-6}$	$-8.15\times 10^{-6}$	$1.36\times 10^{-6}$	$5.43\times 10^{-7}$	$2.15\times 10^{-6}$
TDE + AFISMC (f2)	$-1.38\times 10^{-5}$	$3.16\times 10^{-6}$	$-6.46\times 10^{-6}$	$5.85\times 10^{-5}$	$2.83\times 10^{-5}$	$-5.45\times 10^{-5}$

**Table 9:** The RMS of the control accuracy errors of the target trajectory for case C (δ=3 ms).

Method	$e_{rms}-x$	$e_{rms}-y$	$e_{rms}-z$	$e_{rms}-rx$	$e_{rms}-ry$	$e_{rms}-rz$
TDE + ISMC (sign)	$7.24\times 10^{-5}$	$8.15\times 10^{-5}$	$6.07\times 10^{-5}$	$3.58\times 10^{-4}$	$1.10\times 10^{-3}$	$9.04\times 10^{-4}$
TDE + ISMC (sat)	$6.79\times 10^{-5}$	$7.23\times 10^{-5}$	$6.31\times 10^{-5}$	$3.83\times 10^{-4}$	$1.30\times 10^{-3}$	$7.33\times 10^{-4}$
TDE + AFISMC (f1)	$3.53\times 10^{-5}$	$1.20\times 10^{-5}$	$1.15\times 10^{-5}$	$2.19\times 10^{-5}$	$6.63\times 10^{-5}$	$1.09\times 10^{-4}$
TDE + AFISMC (f2)	$4.75\times 10^{-5}$	$4.94\times 10^{-5}$	$4.79\times 10^{-5}$	$1.98\times 10^{-4}$	$3.06\times 10^{-4}$	$4.49\times 10^{-4}$

**Table 10:** The mean of the control accuracy errors of the target trajectory for case C (δ=5 ms).

Method	$e_{mean}-x$	$e_{mean}-y$	$e_{mean}-z$	$e_{mean}-rx$	$e_{mean}-ry$	$e_{mean}-rz$
TDE + ISMC (sign)	$-2.00\times 10^{-4}$	$5.14\times 10^{-5}$	$3.59\times 10^{-5}$	$7.36\times 10^{-4}$	$-4.36\times 10^{-4}$	$6.22\times 10^{-6}$
TDE + ISMC (sat)	$-2.00\times 10^{-4}$	$4.15\times 10^{-5}$	$5.74\times 10^{-5}$	$1.40\times 10^{-3}$	$-5.08\times 10^{-4}$	$-1.04\times 10^{-5}$
TDE + AFISMC (f1)	$-5.36\times 10^{-6}$	$-2.31\times 10^{-7}$	$1.48\times 10^{-7}$	$4.06\times 10^{-6}$	$4.29\times 10^{-6}$	$-2.19\times 10^{-6}$
TDE + AFISMC (f2)	$-2.48\times 10^{-5}$	$-3.32\times 10^{-6}$	$-2.52\times 10^{-5}$	$1.32\times 10^{-4}$	$4.27\times 10^{-5}$	$-1.16\times 10^{-4}$

**Table 11:** The RMS of the control accuracy errors of the target trajectory for case C (δ=5 ms.)

Method	$e_{rms}-x$	$e_{rms}-y$	$e_{rms}-z$	$e_{rms}-rx$	$e_{rms}-ry$	$e_{rms}-rz$
TDE + ISMC (sign)	$2.52\times 10^{-4}$	$2.24\times 10^{-4}$	$2.00\times 10^{-4}$	$1.60\times 10^{-3}$	$3.90\times 10^{-3}$	$1.20\times 10^{-3}$
TDE + ISMC (sat)	$2.41\times 10^{-4}$	$2.14\times 10^{-4}$	$1.71\times 10^{-4}$	$2.00\times 10^{-3}$	$2.70\times 10^{-3}$	$1.00\times 10^{-3}$
TDE + AFISMC (f1)	$4.71\times 10^{-5}$	$5.27\times 10^{-5}$	$2.18\times 10^{-5}$	$5.12\times 10^{-5}$	$1.22\times 10^{-4}$	$2.48\times 10^{-4}$
TDE + AFISMC (f2)	$1.52\times 10^{-4}$	$1.20\times 10^{-4}$	$1.59\times 10^{-4}$	$4.92\times 10^{-4}$	$5.53\times 10^{-5}$	$7.01\times 10^{-4}$

## Data Availability

The authors confirm that the data supporting the findings of this study are available with in the article.
